# Personalised regional modelling predicts tau progression in the human brain

**DOI:** 10.1371/journal.pbio.3003241

**Published:** 2025-07-21

**Authors:** Pavanjit Chaggar, Jacob W. Vogel, Alexa Pichet Binette, Travis B. Thompson, Olof Strandberg, Niklas Mattsson-Carlgren, Linda Karlsson, Erik Stomrud, Saad Jbabdi, Stefano Magon, Gregory Klein, with the Alzheimer’s Disease Neuroimaging Initiative, Oskar Hansson, Alain Goriely

**Affiliations:** 1 Mathematical Institute, University of Oxford, Oxford, United Kingdom; 2 Clinical Memory Research Unit, Department of Clinical Sciences Malmö, Lund University, Malmö, Sweden; 3 Department of Physiology and Pharmacology, Université de Montréal, Montréal, Quebec, Canada; 4 Centre de Recherche de l’Institut Universitaire de Gériatrie de Montréal, Montréal, Quebec, Canada; 5 Department of Mathematics and Statistics, Texas Tech University, Lubbock, Texas, United States of America; 6 Memory Clinic, Skåne University Hospital, Lund University, Lund, Sweden; 7 Wallenberg Center for Molecular Medicine, Lund University, Lund, Sweden; 8 Wellcome Centre for Integrative Neuroimaging, University of Oxford, Oxford, United Kingdom; 9 F. Hoffmann-La Roche Ltd., Basel, Switzerland; Massachusetts Institute of Technology, UNITED STATES OF AMERICA

## Abstract

Aggregation of the hyperphosphorylated tau protein is a central driver of Alzheimer’s disease, and its accumulation exhibits a rich spatiotemporal pattern that unfolds during the course of the disease, sequentially progressing through the brain across axonal connections. It is unclear how this spatiotemporal process is orchestrated, namely, to what extent the spread of pathologic tau is governed by transport between brain regions, local production, or both. To address this, we develop a mechanistic model from tau PET data to describe tau dynamics along the Alzheimer’s disease timeline. Our analysis reveals longitudinal changes in production and transport dynamics in two independent cohorts, with subjects in the early stage of the disease exhibiting transport-dominated spread, consistent with an initial spread of pathological tau seeds, and subjects in the late stage disease characterized primarily by local tau production. Further, we demonstrate that the model can predict accurately subject-specific longitudinal tau accumulation at the regional level, potentially providing a new clinical tool to monitor and classify patient disease progression.

## Author summary

A mathematical model explains difference between early and late tau protein dynamics in Alzheimer’s, showing stage-specific shifts from transport to local production.

## Abstract 3

The accumulation of aggregated tau shows a complex spatio-temporal pattern during the course of the Alzheimer’s Disease, but how these changes are orchestrated is unclear. This modeling study describes tau protein dynamics in Alzheimer’s disease, showing stage-specific shifts in its local production and transport.

## Introduction

Alzheimer’s disease (AD) is a devastating neurological condition resulting in progressive brain atrophy and cognitive decline. The toxic forms of two proteins, amyloid-β (Aβ) and tau protein (tau) are believed to act in concert to drive AD progression [1, 2]. The pathological roles of these proteins in the human brain during AD have been investigated using positron emission tomography (PET), with radiotracers such as [${\;}^{18}\text{F}$]florbetapir and [${\;}^{18}\text{F}$]flortaucipir allowing for in-vivo quantification of Aβ and tau, respectively [3]. While Aβ tends to be more diffusely present throughout the cerebral cortex [4–7], tau exhibits richer spatiotemporal dynamics, characterised by Braak staging [8]. Braak staging describes the trajectory of toxic tau, starting from the entorhinal cortex and sequentially progressing to the limbic regions, the basal temporal lobes, the broader association cortex, and finally the primary sensory cortex. This staging pattern has been validated using tau PET imaging [9–11] and has been shown to be highly correlated with atrophy and cognitive decline [12, 13], however, the mechanism for how tau staging is orchestrated remains unclear.

Growing evidence suggests that the progression of AD depends on two distinct factors: 1) the local production of toxic proteins; 2) the transport of toxic proteins throughout the brain. However, it has yet to be determined to what extent these factors contribute to the progression of AD and whether their contributions change over time. There is now substantial evidence that tau propagation follows a prion-like mechanism, progressively forming toxic oligomeric seeds and neurofibrillary tangles through an autocatalyic production process [14, 15]. The prion-like nature of tau has been demonstrated with transgenic animal models in which cortical injections of tau seeds induce the formation of tau aggregates that grow in concentration over time at the injection site and surrounding areas [16, 17]. In 2012, studies by Liu *et al*. and de Calignon *et al*. showed that transgenic mice overexpressing pathological human tau in the entorhinal cortex exhibit accumulation of tau aggregates and that tau invades axonally connected regions through transsynaptic transport to form seeds in otherwise healthy regions [18, 19]. Prion-like aggregation and axon-based tau transport have also been demonstrated in human postmortem studies [20] and in vivo studies using structural connectome-based models of tau PET capable of reproducing observed tau aggregation and spread [21–26]. In a recent investigation, Meisl *et al*. analysed multimodal tau data from Braak stage 3 onward and showed that tau production, not transport, is the main contributor of tau progression [27]. However, the study does not account for the spatial progression across individual brain regions or estimate dynamics across the full AD progression timeline. Unanswered questions remain about whether there are changes in tau production and transport rates over time and whether the balance of these two processes changes along the disease timeline. To address these outstanding questions, we develop a whole brain model capable of accurately describing longitudinal tau PET data and conduct a multicohort study to analyse tau dynamics across the full disease progression timeline.

To answer questions about temporal changes in AD tau dynamics in the human brain, an accurate and reliable model of longitudinal tau observations is needed. In the past decade, there have been numerous efforts to use mathematical models to better understand the spatiotemporal properties of AD pathology, ranging from linear diffusion models of tau [21, 22] to infinite-dimensional spatiotemporal models of toxic protein aggregation [28]. Each of these models makes different assumptions about the physical mechanisms of tau spread; however, there has not been a unifying effort to rigorously compare commonly used models to identify which are best able to accurately describe longitudinal tau PET observations. In addition, the models currently described in the literature do not account for regional variations in tau dynamics, which has been shown to influence tau progression [29–31] and are incapable of predicting longitudinal changes at the regional level. Here, we present a novel model that provides a qualitative account of regional vulnerability and its effect on tau progression. Using a previously developed Bayesian pipeline for longitudinal tau modelling [26, 32], we perform hypothesis-driven model selection on a family of common models from the AD modelling literature, including a new model that accounts for regional dynamics. We show that models that rely only on network diffusion or homogeneous tau production dynamics are not sufficient to model regional longitudinal data, whereas models accounting for regional variations in tau dynamics are able to accurately model longitudinal tau observations at a regional level. We validate our model by showing that it can *forecast* regional rates of tau accumulation over time for individual patients. The combination of these methods provides a novel pipeline for analysing and understanding longitudinal tau data, allowing us to compare changes in disease dynamics throughout the AD timeline and predict subject-specific, region-specific changes in tau over time.

We combine state-of-the-art modelling and inference methods with longitudinal tau PET data from two independent cohorts to address the outstanding question of how tau transport and production drive AD progression. To determine whether there are changes in tau transport and production rates during the progression of AD, we apply our model to three groups representing different stages of the AD timeline [33, 34]: (i) ${\text{A}}^{+}{\text{T}}^{+}$: A_𝛽_ positive, tau positive; (ii) ${\text{A}}^{+}{\text{T}}^{-}$: A_𝛽_ positive, tau negative; (iii) ${\text{A}}^{-}{\text{T}}^{-}$: A_𝛽_ negative, tau negative. We show that tau transport is faster in early stage disease (${\text{A}}^{+}{\text{T}}^{-}$), and that there are primary and secondary increases in tau production dynamics throughout the disease timeline. Finally, we validate these results on an independent dataset, BioFINDER-2, using a different tau tracer, on which the same results are obtained, further showing that the model and results are robust and generalisable between datasets and the choice of tau tracer.

## Results

### Deriving a generative model of tau dynamics

We first extend previous work [28, 35, 36] to develop a mechanism-based model of tau dynamics in the human brain that can be calibrated using tau PET data.This model, called the *local FKPP model* (which is a network version of the well-known continuous Fisher-Kolmogorov–Petrovsky–Piskunov equation) and derived in full detail in the Methods section, is given by a set of non-linear ordinary differential equations on a structural connectome network of *R* nodes for the variables $s_{i}=s_{i}(t)$ for i=1,⋯,R, representing tau SUVR in different regions of interest:

dsidt=−ρ∑j=1Rℒij(sj−s0,j)⏟transport+∑j=1Rα(si−s0,i)[(s∞,i−s0,i)−(si−s0,i)]⏟production,i=1,…,R. (1)

The first term represents the contribution of tau transport between brain regions through a graph Laplacian ℒ and uniform rate ρ for the *i*-th region, consistent with previous work [21, 22, 24, 36]. The second term represents prion-like production at the *i*-th region with uniform rate α [26, 36]. While we refer to α as the production parameter throughout this manuscript, we note that its value encapsulates a broad number of processes that effect change in tau SUVR, such as tau clearance, aggregation, fragmentation and atrophy effects. Therefore, the α parameter represents the net change in tau PET subject to the numerous processes that govern its progression and can be negative if processes such as atrophy of clearance outweigh tau production. The local FKPP model describes future changes in tau SUVR as resulting from a combination of transport through axonal connections and prion-like production. In this study, regions of interest are given by the 68 cortical regions of the Desikan-Killiany (DK) atlas, in addition to bilateral hippocampus and amygdala, therefore, there are *R* = 72 nodes in the connectome model. We introduce two novel parameter vectors, regional baseline values, *s*_0,*i*_, and carrying capacities $s_{\infty ,i}$, that represent a healthy state and a late stage AD state, respectively, *for i* = 1 … *R* which add information about regional variations in production dynamics. To make the relationship to regional variability in production rates of tau more clear, consider a change of variables to $q_{i}=\left(s_{i}-s_{0,i}\right)/\left(s_{\infty ,i}-s_{0,i}\right)$ for i=1⋯R, then Eq (1) becomes a standard (network) FKPP equation:

$$\frac{dq_{i}}{\text{d}t}=-\rho \sum_{j=1}^{R}{\hat{ℒ}}_{ij}q_{j}+{\hat{\alpha }}_{i}q_{i}\left(1-q_{i}\right)\;i=1,\ldots ,R,$$ (2)

with $\hat{\alpha }=\alpha {𝐪}_{\infty }$ and $\hat{ℒ}={𝐪}_{\infty }^{-1}ℒ{𝐪}_{\infty }$, with ${𝐪}_{\infty }={𝐬}_{\infty }\;-\;{𝐬}_{0}$. In this form, regional tau evolves between [0,1] with a rate that depends on the difference between regional SUVR baseline values and carrying capacities, with larger differences equating to faster regional tau production, making the notion of regional vulnerability more explicit.

**Fig 1:**
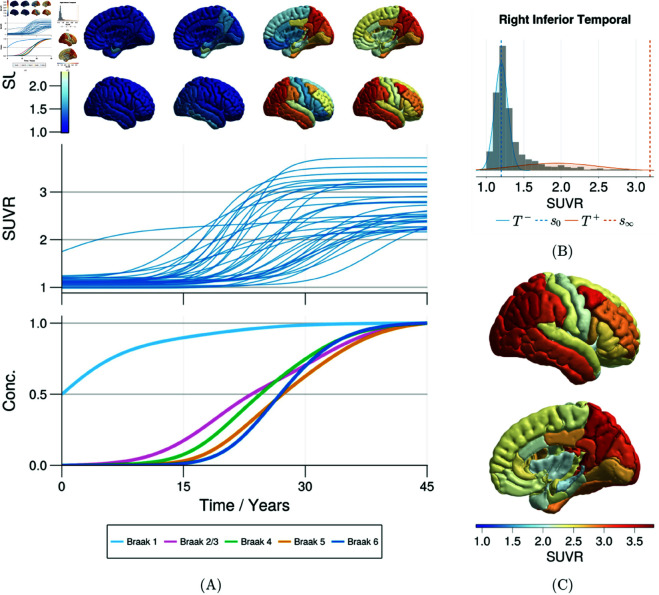
Simulated transport and production dynamics in the local FKPP model. (A) Simulation from the local FKPP model using carrying capacities derived from Gaussian mixture models (shown in Fig 1B). Simulations are initialised with a seed value of $(s_{0,i}\;+\;s_{\infty ,i})/2$ in the bilateral entorhinal cortex, i={27,63}, with ρ=0.025 and α=0.25. Each line in the middle panel represents the SUVR trajectory of one DK atlas brain region. Values at time points t={0,15,30,45} years are projected onto a cortical rendering in the top panel. Each line in the bottom panel represents concentration averaged over Braak regions, after rescaling simulated SUVR as $q_{i}=\left(s_{i}-s_{0,i}\right)/\left(s_{\infty ,i}-s_{0,i}\right)$. (B) Two component Gaussian mixture model fit to a multi-cohort tau PET dataset [24] and data from ADNI for right inferior temporal lobe. Baseline values for each region are taken as the mean of regional ${\text{T}}^{-}$ distribution and the carrying capacity as the 99-th percentile of the regional *T*^ + ^ distribution, and these are used to simulate the model throughout this paper. (C) Right hemisphere cortical rendering of the SUVR carrying capacities as determined through Gaussian mixture modelling.

The regional parameters are derived from the Gaussian mixture modelling approach used in [24], in which a two-component Gaussian mixture model is used to describe healthy and pathological tau SUVR distributions (see Methods for more details). An example of this is shown in Fig 1B for the right inferior temporal lobe, and the carrying capacities for the right hemisphere are shown in Fig 1C. Since these parameters are estimated from tau PET, they also encode specific features of the tracer, such as regional differences in tracer uptake, specificity to 3R/4R tau pathology, on-target binding and off-target binding, and therefore allow us to model tau SUVR directly. An example of simulated regional trajectories is shown in Fig 1A, encapsulating the effect of both transport and production. A consequence of the variation of carrying capacities is that the regional production rates also vary between regions, as seen in the middle panel of Fig 1A, providing a qualitative account of regional vulnerability. Using the rescaled local FKPP model Eq (2), we can also track the simulated concentration of tau in Braak regions, shown in the bottom panel of Fig 1A, and we see that the model correctly predicts the invasion of tau into Braak regions. The ability to account for these regional variations extends previous models with homogeneous dynamics across regions [23, 36] providing a picture of tau progression that is more consistent with observed tau staging.

### Regional heterogeneity is necessary for longitudinal prediction

To determine whether the local FKPP is capable of fitting observed AD trajectories, we compare it to tau PET data. For comparison, we also consider simpler models that can be obtained from the local FKPP model Eq (1), namely *the global FKPP model*, Eq (12), obtained by assuming that none of the parameters vary locally, *the diffusion model* Eq (11) obtained by taking α=0 in Eq (1), and *the logistic model* Eq (10) obtained by neglecting transport between regions, ρ=0.

**Fig 2:**
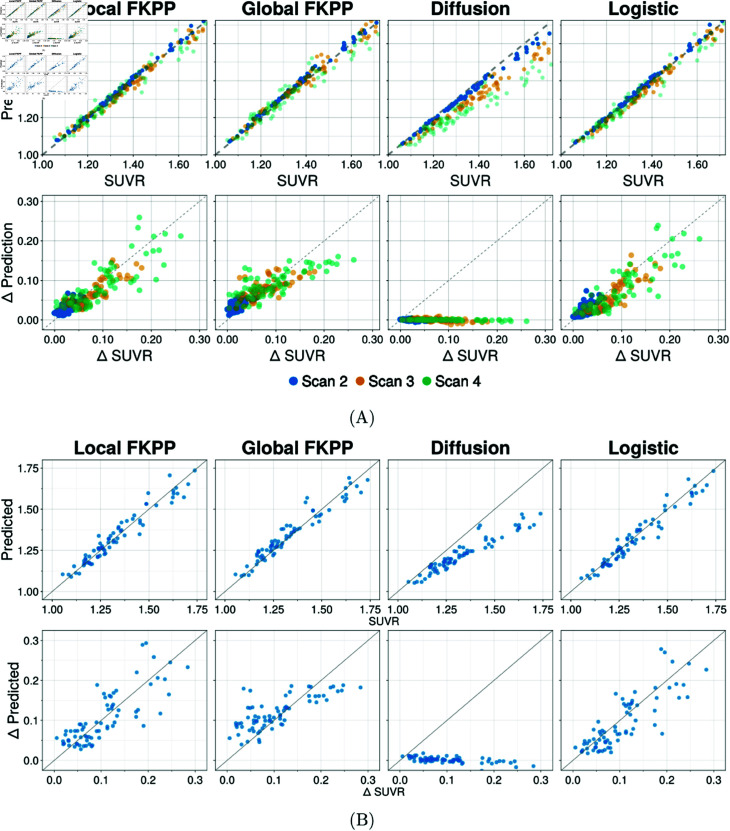
Model fit for in-sample and out-of-sample data. (A) Goodness of fit for four models, local FKPP, global FKPP, diffusion and logistic. For all panels, each point represents a region in the connectome model, averaged over subjects per scan. Top row shows estimated vs observed SUVR values. Bottom row shows estimated change vs observed change in SUVR. The local FKPP, global FKPP and logistic models provide the best fit to longitudinal data, while the diffusion model is unable to capture longitudinal change since it does not describe production. (B) Out-of-sample fits for four models of proteopathy. Top row: predicted vs observed out-of-sample SUVR. Bottom row: predicted change vs observed change from first in-sample scan to last out-of-sample scan. Each point represents a region in the connectome model, averaged over subjects.

We use hierarchical Bayesian inference to calibrate each model to tau PET data, allowing us to quantify whether the model parameters can be identified from patient data and provide bounds on uncertainty for group-level and individual-level parameters. We use tau PET data from ADNI, selecting A$\beta^{+}$ subjects who have at least three scans and are *T*^ + ^ in the medial temporal or lateral temporal lobe (see Methods for details). We employ two metrics to compare models, the Bayesian information criteria (BIC) to measure accuracy against in-sample data and the expected log predictive density (ELPD) to measure out-of-sample predictive accuracy, both provided in Table 1. Fig 2A shows the in-sample longitudinal fit for each of the four models and Fig 2B shows the regionally averaged out-of-sample longitudinal fit.

**Table 1:** Assessment of model fit using the Bayesian information criteria, BIC, and the expected log predictive density, ELPD. Lower values are considered better for the BIC and higher values are better for the ELPD. The local FKPP model performs best in both metrics.

	Local FKPP	Global FKPP	Diffusion	Logistic
BIC	–33889.2	–32823.1	–20539.1	–33138.7
ELPD	638.22	622.68	–175.579	607.14

The local FKPP performs best for both in-sample fit and out-of-sample predictive accuracy, followed by the logistic model for in-sample fit and the global FKPP model for out-of-sample fit. The results show that the diffusion model is not suitable for longitudinal modelling of tau PET data, clearly shown in Fig 2A and 2B, since there is no mechanism for tau production or clearance and therefore the total concentration is conserved. The global FKPP model can capture changes in tau load, but it cannot describe the regional heterogeneity in tau production. The deficiencies in production dynamics of the diffusion and global FKPP models are addressed with the local FKPP model and the logistic model and the ability of these models to accurately describe longitudinal tau PET underlines the importance of including regionally specific production rates. The logistic model is capable of describing the trajectory of tau PET despite not being able to capture the transport of tau through the structural connectome, suggesting that ${\text{A}}^{+}{\text{T}}^{+}$ subjects may already have widespread invasion of tau seeds. However, the logistic model under-fits changes in SUVR, particularly in the low SUVR range. The error in model fit relative to the final in-sample scan is shown in S1 Fig for the local FKPP, global FKPP and logistic model. The residual analysis shows that the logistic model is prone to underestimate SUVR (mean error=−0.02, s.d.=0.02), particularly for lower SUVR ranges, as shown in S1 Fig, while the error in the local FKPP (mean error=−0.01, s.d.=0.02) and global FKPP (mean error=0.005, s.d.=0.03) is more balanced between regions. This indicates that initial tau deposition may be driven by transport between regions. Additionally, the global FKPP has a higher variance in predictions, often either overestimating or underestimating SUVR as a result of regionally homogeneous dynamics. Overall, the data support the use of the local FKPP model, as evidenced by its being the most capable of describing in-sample and out-of-sample data, while also capturing the role of both tau transport and local tau production.

### Local model forecasts regional tau progression

**Fig 3:**
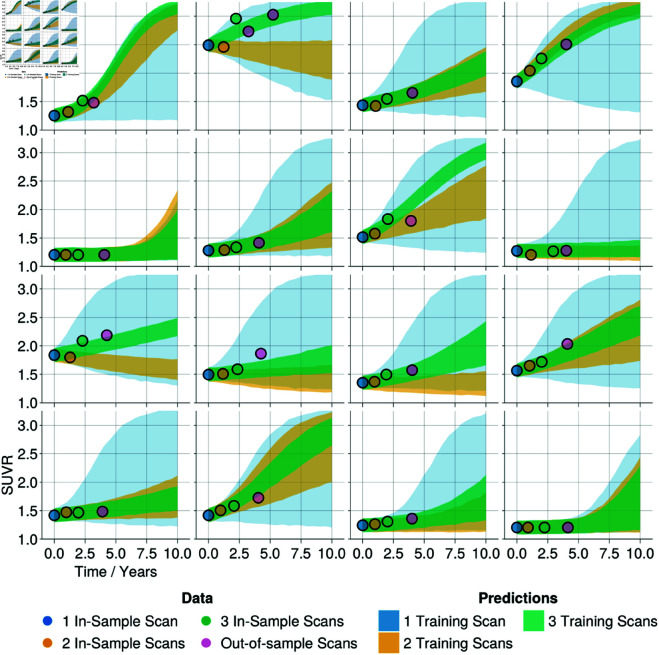
Out-of-sample fit and posterior predictive plots for the left inferior temporal region. The local FKPP model was iteratively calibrated to a ${\text{A}}^{+}{\text{T}}^{+}$ ADNI cohort with 41 in-sample subjects and 16 test subjects. Three iterations were run where for each iteration an additional scan from the test subjects were included, starting with a single scan. Posterior predictive trajectories for left inferior temporal lobe are shown for each iteration (neglecting observation noise). In the above figure, each panel represents one of the 16 test subjects. Each point represents a data point added for a training iteration; trajectories are colour matched to correspond to the number of longitudinal data points included for training.

A major possible benefit of the mathematical modelling of AD lies in its application to clinical and pharmacological research, particularly by predicting the progression of the patient’s disease. Here, we show that the local FKPP model can be used to accurately *forecast* the trajectory of tau PET. We divide the ADNI ${\text{A}}^{+}{\text{T}}^{+}$ cohort into train and test cohorts. We define a fixed training set comprising *n* = 41 subjects who have three longitudinal scans. For the remaining *N* = 16 subjects with more than three longitudinal scans, we perform inference three times, each time adding an additional scan to the training set, starting with a single scan. The resulting posterior predictive trajectories for the left inferior temporal lobe are shown in Fig 3. In S2 Fig we provide the posterior predictive trajectories without observation noise, highlighting the uncertainty in model parameters. The results show that a single scan is often insufficient to provide meaningful forecast accuracy, despite benefiting from information pooled across subjects in the hierarchical Bayesian model. This greatly improves with the addition of a second data point; however, in some cases this produces inaccurate forecasts of future data if there is a decrease in tau SUVR, perhaps due to atrophy. With the addition of a third data point, the results generally converge with low uncertainty and accurately forecast future observations. Similar results are shown in the supplementary information for the entorhinal cortex S3 and S4 Fig. The results suggest that a more constrained approach which more heavily weights the effect of population priors on forecasts may prove fruitful in circumstances in which there is insufficient data to capture an individual’s trajectory. Nonetheless, these results demonstrate the power of a simple model based on key biological priors (namely the connectome, regional baseline values, and regional carrying capacities) and two free parameters to forecast the regional progression of tau PET that may provide benefit to clinical and pharmaceutical researchers.

### Early AD progression is driven by tau transport

Next, we sought to determine whether there are any changes in tau production and transport dynamics across the AD progression timeline. To do so, we use two cohorts of tau PET data, ADNI and BioFINDER-2 (BF2), each divided into three groups, ${\text{A}}^{+}{\text{T}}^{+}$, ${\text{A}}^{+}{\text{T}}^{-}$, ${\text{A}}^{-}{\text{T}}^{-}$, representing different stages of AD. Since BF2 uses a different tau PET radiotracer, we rerun the Gaussian mixture modelling analysis to recover the tracer-specific baseline and carrying capacities. We then apply the NUTS sampling algorithm to the hierarchical Bayesian model to obtain samples from the posterior distribution, Eq (17), providing distributions for model parameters at the population level and individual level.

**Fig 4:**
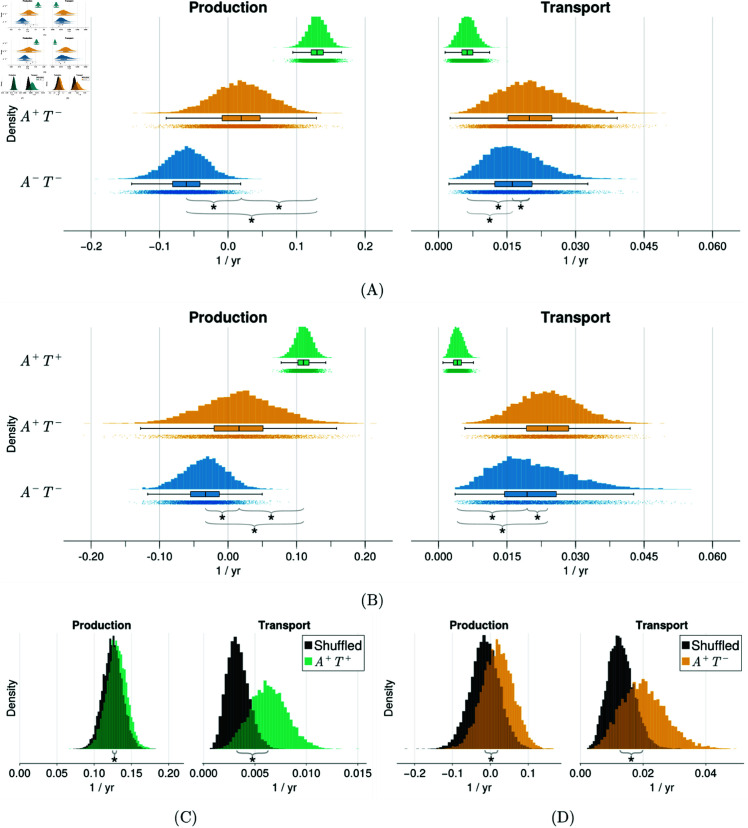
Inferred population level parameters using ADNI and BF2 tau data. (A) & (B) Population production and transport parameters across ${\text{A}}^{+}{\text{T}}^{+}$, ${\text{A}}^{+}{\text{T}}^{-}$ and ${\text{A}}^{-}{\text{T}}^{-}$ groups for ADNI (A) and BF2 (B) tau PET data. (C) & (D) Inferred population production and transport parameters from spatially shuffled data (shown in grey) compare to inferred distributions from true data for ${\text{A}}^{+}{\text{T}}^{+}$ (C), ${\text{A}}^{+}{\text{T}}^{-}$ (D) ADNI groups. Asterisks between groups denotes distributions are significantly different (*p* < 0.01), tested using the Mann-Whitney U test. Data underlying this figure can be found at https://doi.org/10.5281/zenodo.15389493

The distributions of the population parameters for the ${\text{A}}^{+}{\text{T}}^{+}$, ${\text{A}}^{+}{\text{T}}^{-}$, ${\text{A}}^{-}{\text{T}}^{-}$ groups are shown in Fig 4A for ADNI and Fig 4B for BF2 and are summarised in Table 2. Additionally, all individual level posterior distributions are provided in S5 Fig for ADNI and S6 Fig for BF2. The posterior distributions between cohorts are qualitatively the same, with changes likely reflecting differences in cohort and tracers. The inferred parameters show an increase in the transport rate for the ${\text{A}}^{+}{\text{T}}^{-}$ group compared to the ${\text{A}}^{+}{\text{T}}^{+}$ and ${\text{A}}^{-}{\text{T}}^{-}$ groups, suggesting that tau spreads more easily between regions during the early stages of the disease and is minimal in the later stages of AD. The inferred posterior distributions for the production parameter show a progressive increase in the production rate along the disease timeline, with a primary increase from the ${\text{A}}^{-}{\text{T}}^{-}$ to the ${\text{A}}^{+}{\text{T}}^{-}$ group and a secondary increase from the ${\text{A}}^{+}{\text{T}}^{-}$ group to the ${\text{A}}^{+}{\text{T}}^{+}$ group. The negative production rate for the ${\text{A}}^{-}{\text{T}}^{-}$ group indicates that the signal, on average, decreases. This could reflect changes in noise due to off-target, non-specific binding, or atrophy from non-AD related neurodegeneration. Overall, these results suggest that in early AD (${\text{A}}^{+}{\text{T}}^{-}$) tau begins in a transport-dominated phase (ρ>α) and then switches to a production-dominated phase (α>ρ) later in AD (${\text{A}}^{+}{\text{T}}^{+}$).

**Table 2:** Summary of the inferred posterior distributions for the population parameters in ADNI and BF2, where $\rho_{\mu }$ is the average of population transport parameter, $\rho_{\sigma }$ is the standard deviation of the population transport parameter, $\alpha_{\mu }$ is the average population production parameter, $\alpha_{\sigma }$ is the standard deviation of the population production parameter. Parameters are shown for each of the ${\text{A}}^{+}{\text{T}}^{+}$, ${\text{A}}^{+}{\text{T}}^{-}$ and ${\text{A}}^{-}{\text{T}}^{-}$ groups.

	Group	$\rho_{\mu }$	$\rho_{\sigma }$	$\alpha_{\mu }$	$\alpha_{\sigma }$
ADNI	${\text{A}}^{+}{\text{T}}^{+}$	0.007	0.011	0.130	0.100
	${\text{A}}^{+}{\text{T}}^{-}$	0.020	0.043	0.019	0.232
	${\text{A}}^{-}{\text{T}}^{-}$	0.017	0.045	–0.061	0.194
BF2	${\text{A}}^{+}{\text{T}}^{+}$	0.004	0.008	0.110	0.088
	${\text{A}}^{+}{\text{T}}^{-}$	0.024	0.024	0.015	0.221
	${\text{A}}^{-}{\text{T}}^{-}$	0.021	0.083	–0.034	0.205

To test where the parameter distributions reflect meaningful dynamics present in the data and are not as a result of statistical patterns or noise, we rerun the analysis on the ${\text{A}}^{+}{\text{T}}^{-}$ and ${\text{A}}^{+}{\text{T}}^{+}$ groups using spatially shuffled data. A comparison of the posterior distributions obtained shuffled data and those obtained from true data are shown in Fig 4C and 4D. Note that we only spatially shuffle the data and therefore expect minimal changes to the estimated production parameters. We note a marked difference in the estimated transport dynamics in both the ${\text{A}}^{+}{\text{T}}^{-}$ and ${\text{A}}^{+}{\text{T}}^{+}$ groups between the true and shuffled data, confirming that the dynamics present in the data are not a consequence of statistical patterns or noise in the data, but represent tau dynamics measured through PET.

To examine whether the negative production rates observed in Fig 4 are a result of regional atrophy, we rerun the analysis in the ADNI cohort with partial volume correction applied [37, 38]. The population-level posterior distributions resulting from this analysis are shown in Fig A in S1 Text. The results are qualitatively the same as those obtained using non-PVC data Fig 4, with increases in tau production along the AD progression timeline and a faster transport rate in ${\text{A}}^{+}{\text{T}}^{-}$ subjects compared to ${\text{A}}^{+}{\text{T}}^{+}$ and ${\text{A}}^{-}{\text{T}}^{-}$ subjects. However, using PVC data results in an increased production rate for the ${\text{A}}^{-}{\text{T}}^{-}$ and ${\text{A}}^{+}{\text{T}}^{-}$ groups, largely eliminating the negative production rate observed with non-PVC data. Furthermore, in S7 Fig we show the correlation between the longitudinal change in regional cortical thickness and longitudinal change in regional SUVR for the ${\text{A}}^{+}{\text{T}}^{+}$, ${\text{A}}^{+}{\text{T}}^{-}$ and ${\text{A}}^{-}{\text{T}}^{-}$ groups. We observe a positive correlation between change in cortical thickness and change in SUVR in the ${\text{A}}^{-}{\text{T}}^{-}$ group but not in the ${\text{A}}^{+}{\text{T}}^{-}$ or ${\text{A}}^{+}{\text{T}}^{+}$ groups, indicating that the decreasing SUVR in the ${\text{A}}^{-}{\text{T}}^{-}$ group is a result of regional atrophy. Overall, these analyses suggest that the negative production rate observed in Fig 4 primarily results from brain atrophy outpacing tau production, resulting in a net reduction of SUVR.

Additionally, we rerun the analysis using an eroded white matter reference region, which has been suggested as a more effective reference region for longitudinal analysis [39]. The posterior distributions for this dataset are shown in Fig A in S1 Text. The results show a similar transport effect to Fig 4. However, there are significant differences in the inferred production parameters. The ${\text{A}}^{+}{\text{T}}^{-}$ and ${\text{A}}^{+}{\text{T}}^{+}$ groups both display higher production than the ${\text{A}}^{-}{\text{T}}^{-}$ group, however, there is no significant difference between the ${\text{A}}^{+}{\text{T}}^{-}$ and ${\text{A}}^{+}{\text{T}}^{+}$ groups. After further analysis, we believe this effect is due to positive correlations in the reference region and the target SUVR that reduce group differences and limit interpretation of the longitudinal analysis (see Fig B in S1 Text for analysis).

The large parcels present in the DK atlas may affect the detail in which production and transport dynamics are present in the data, since data is averaged over large parcels with heterogeneous volumes. To address this, we rerun the analysis using the Schaefer-200 atlas [40] provided in the ENIGMA toolbox [41]. The population-level model parameters for the ${\text{A}}^{+}{\text{T}}^{+}$, ${\text{A}}^{+}{\text{T}}^{-}$, and ${\text{A}}^{-}{\text{T}}^{-}$ groups are shown in Fig C in S1 Text. The transport parameters between groups are qualitatively similar to those shown in Fig 4, further supporting an initial phase of accelerated tau transport. The production parameter was higher for the ${\text{A}}^{+}{\text{T}}^{+}$ group compared to the ${\text{A}}^{+}{\text{T}}^{-}$ and ${\text{A}}^{-}{\text{T}}^{-}$ groups, consistent with the DK atlas. However, there is no longer a substantial increase from ${\text{A}}^{-}{\text{T}}^{-}$ to ${\text{A}}^{+}{\text{T}}^{-}$, with the two groups showing similar tau production rates, possibly as a result of greater sensitivity to tau-related atrophy effects due to smaller parcel sizes.

The transport dominated phase of the early AD subjects (${\text{A}}^{+}{\text{T}}^{-}$) supports evidence showing tau seeds are present throughout the cortex before symptom onset [20, 42] and, together with the small role of transport in the ${\text{A}}^{+}{\text{T}}^{+}$ groups, helps explain the strong performance of the logistic model in Fig 2. Overall the results reveal temporal changes in the dynamics of tau progression, with an initial transport dominated phase, perhaps in which seeds are deposited around the cortex, followed by a production dominated phase indicative of secondary tauopathy, likely due to spatial colocalisation with Aβ catalysing tau production.

## Discussion

We have derived a physics-based generative model to describe tau PET data in terms of underlying tau dynamics and applied it data from ADNI and BF2 to understand how it compares to other models present in the literature and how it can inform us about tau transport and production dynamics in the human brain. We have shown that this model can forecast accurately longitudinal regional tau PET progression in AD subjects. Furthermore, by performing inference across different patient groups across the AD disease timeline, we uncover temporal changes in transport and production dynamics, showing an initial transport dominated phase associated with early AD (${\text{A}}^{+}{\text{T}}^{-}$) and tau seeding, followed by an accelerated production dominated phase (${\text{A}}^{+}{\text{T}}^{+}$ onwards) indicative of secondary tauopathy.

Several studies have proposed different models of proteopathy in AD, a key difference between them being descriptions of the tau production process, which vary widely in complexity [22, 24–28, 43]. Here we present a parsimonious model of tau progression that relies on regionally specific carrying capacities and show through model selection (Fig 2) that it is capable of outperforming other models proposed in the literature. A possible cause of regional heterogeneity in carrying capacities is heterogeneity in regional risk factors that promote tau proliferation, the most likely of which is Aβ. Aβ has its own spatial topography within AD patients, being particularly present throughout the fronto-partietal-temporal default mode network and stimulating neuronal hyper-activation [11, 44, 45]. The presence of Aβ will have a two-fold effect on tau dynamics, first through a catalysing effect on tau production [46–48] and second through the promotion of activity-dependent spread and production through functional networks [49, 50]. In Thompson *et al*. 2020, we formulated a model describing the dynamic interaction between Aβ and tau, that predicts an increase in carrying capacities based on Aβ concentration [51], however, further work toward simplifying the model will be necessary before it can be used for inference with patient data.

Another key set of factors that contribute to regional vulnerability are genetic markers. It has already been shown in mice models of AD that gene expression patterns can inform tau spread [31] and human models of Parkinson’s disease have shown how gene expression patterns can inform regional vulnerability to create a model of toxic protein spread in Parkinson’s disease [52]. There are several candidate genes for modelling regional vulnerability in AD, most notably the microtubule association protein tau (MAPT), as a proxy for relative baseline tau vulnerability [53] and apolipoprotein-E (APOE) for those patients with the APOEϵ4 mutation [29, 54, 55]. Although there are many other candidate genes that can influence regional vulnerability, care should be taken to avoid creating overparameterised models. In summary, while the work here provides compelling evidence for the need for regional vulnerability, further work should seek to explain the mechanisms through which regional carrying capacities emerge from a culmination of regional risk factors, such as Aβ deposition and gene expression patterns.

There is extensive evidence of tau transport and production throughout the brain, however, it has not yet been determined whether one of these processes dominates the other and whether their relative contributions to disease progression change over time. To this end, we sought to determine whether inferred parameters of our model change in groups across the disease timeline. We find that during the early stages of the disease (${\text{A}}^{+}{\text{T}}^{-}$), when there is a low concentration of tau in the medial temporal lobe, tau dynamics are transport-dominated but become production-dominated later in the disease. This supports previous work by Meisl *et al*. [27] who show through an analysis of multiple datasets and methods of tau quantification that tau dynamics are production dominated from Braak stage 3 onwards. This is consistent with our work, considering individuals who are positive on tau PET in early Braak stage regions may already show fairly advanced Braak stages at autopsy [56] analogous to individuals at middle Braak stages used by Meisl *et al*. 2021. These results suggest that, in early AD, tau seeds invade connected regions from the medial temporal lobe, but the overall concentration does not grow substantially. Only in later stages of AD is the spread production-dominated and driven by fast increases in concentration gradients, leading to progressive Braak-like staging. These results also support the utility of the logistic production model in being able to describe longitudinal ${\text{A}}^{+}{\text{T}}^{+}$ data (Fig 2A and 2B), since seeds would already be densely present around the cortex and progression is driven by tau production. The results are also consistent with experimental evidence showing that tau seeds are present before tau pathology [20, 42].

Together the results indicate an intrinsically spatiotemporal process, with variations in both tau transport and production along the AD timeline. Our results suggest that there is an initial transport-dominated tauopathy that results in tau seeds spreading from the medial temporal lobe to axonally connected regions. This initial phase is followed by a secondary production-dominated tauopathy with accelerated regional accumulation and slower transport, capable of reproducing sequential Braak like staging such as in Fig 1A. This contrasts to the largely temporal process of Aβ, described by [57], where initially Aβ is present throughout the brain, but increases in concentration occur at different rates due to regional vulnerabilities. Our results suggest that the early period of AD during which tau is more easily transported between brain regions may be a critical time for intervention. Many immunotherapies currently being developed act on extracellular tau [58] and should therefore interrupt tau transmission through the extracellular space of the synaptic junction. If AD is a consequence of first tau spread and then tau production, it will be crucial that these immunotherapies are administered early in the AD process to halt the widespread transmission of tau before accelerated local production can occur. In contrast, therapies that act to reduce intracellular tau concentration should be effective in slowing AD progression throughout the AD continuum, regardless of whether widespread tau transmission has occurred [59].

This work presents a step forward in whole brain tau modelling, however, there are still many obstacles that are not addressed here. There are limitations that pertain to the sparsity and quality of longitudinal data. In this work, we fix a number of parameters to ensure the practical identifiability of the models given the available data. In particular, we fix baseline values and carrying carrying in the dynamical system, and subject initial conditions in the probabilistic model. By fixing baseline values and carrying capacities, we are unable to determine whether these also undergo dynamical changes. This also limits the direct application of the model to other tauopathies that exhibit different tau PET profiles. A related limitation is the particular choice of parametrisation for regional vulnerability. Here, we have chosen to parametrise regional vulnerability as changes in regional carrying capacities scaled by a single free parameter. Future work should investigate whether more expressive models, such as regional free parameters or dynamically changing parameters influenced by other risk factors, such as amyloid, are more informative about longitudinal biomarker data. Additionally, there is considerable uncertainty regarding the level of tau pathology that is detectable in ${\text{A}}^{-}{\text{T}}^{-}$ and ${\text{A}}^{+}{\text{T}}^{-}$ groups, that limits interpretation and may preclude accurate forecasting of tau progression.

Second, there are limitations related to the scale at which we are modelling. While the model we present here is derived from a physics-based model, the model reduction comes at the cost of a loss in mechanistic insight into precise transport and production mechanisms. This will remain a hard limitation while we work with macro-scale brain data. In addition, tau PET data is intrinsically limited by resolution, inability to detect early changes, and non-specific and off-target binding sources, collectively providing a source of uncertainty that affects parameter identifiability of intricate processes such as transport. Therefore, while the modelling results suggest changes to transport and production across the AD continuum, our conclusions are limited by the nature of PET measurements and require experimental validation. Given this limitation we are cautious about over-interpreting the model parameters. Notably, the production parameter, α, represents a balance between several factors, including biological factors such as tau clearance, production, aggregation, fragmentation, and volume effects. Therefore, in the ${\text{A}}^{-}{\text{T}}^{-}$ group, where there is a largely negative production rate, this indicates that the clearance of tau or volume effects dominates the tau production process. Conversely, positive tau production rate in the ${\text{A}}^{+}{\text{T}}^{+}$ group indicates tau production outweighs other factors that may decrease the tau PET signal. In both cases, the exact mechanism of tau change remains unclear. Further work should seek to understand precisely how the changes in signal arise. A potential avenue to address this limitation will be the development of multi-scale models that rely on in-vitro or animal studies for calibration and permit macro-scale reduced order models.

Third, as longitudinal scans are added to inference, the overall uncertainty decreases, as shown in Figs 3, S2, S3 and S4, and does not always accurately capture out-of-sample regional data points. This likely results from the assumption of independent and identically distributed noise across subjects, scans and regions. As a result of the sequential staging of tau, there are likely to be more regions that have low signal and low longitudinal change and therefore the grouped noise parameter will be driven by these regions and underestimate noise in regions of high tau production. Future work should incorporate more specific noise models that account for changes in observation noise with the amount of signal, for example, a multiplicative noise model that assumes that noise scales proportionally to signal. Finally, and perhaps most importantly, the model presented here has limited application to real-world clinical data, since most patients are not monitored with longitudinal tau PET and would therefore lack the necessary data with which to calibrate the model. Therefore, while the model can provide useful mechanistic insights, it may not provide immediate clinical utility. We envisage two main applications of the current work. First, to provide motivation for experimental work into understanding changes in tau spreading in-vivo across the AD timeline. Second, direct applications to pharmaceutical trials, in which longitudinal tau PET is more readily available, to inform optimal intervention periods and testing patient trajectories against model predictions. The limitation on data availability may be eased by future work to incorporate fluid biomarker data into the modelling pipeline, as a less invasive and more readily available measure of tau burden. Fluid biomarkers can provide valuable information on tau production levels in-vivo [60, 61] that could be used to calibrate simplified spreading models such as the logistic model presented here, or similar asymptotic approximation to the local FKPP model that require only a production parameter [62].

The primary contribution of this work has been to provide a parsimonious account of regional tau dynamics in AD. Future work should seek to build on this, adding more information and data to probe the unexplained dynamics in AD. Most pressingly, these include dynamical interactions between Aβ and tau in a sufficiently simple way to accommodate the ability to perform inference with patient data. Furthermore, the study sheds light on potential avenues of clinical investigation of anti-tau therapies by showing how targeting different tau processes (transport or local production) at different times during the AD continuum may be essential for effective intervention.

## Methods

### Data processing

We use data from the Alzheimer’s Disease Neuroimaging Initiative (adni.loni.usc.edu). ADNI is a public-private partnership with the aim of using serial biomarkers to measure the progression of AD. For up-to-date information, see www.adni-info.org. We download fully processed tau PET and magnetic resonance image (MRI) data, summarised as standardised uptake value ratios (SUVR) and volumes for each of the regions in the Desikan-Killiany (DK) atlas. We renormalise individual SUVR using an inferior cerebellum SUVR reference region. Amyloid status for ADNI subjects was also downloaded from ADNI and used to classify subjects, with Aβ positivity requiring cortical summary florbetapir SUVR > 0.78 or florbetaben SUVR > 0.74, using the composite reference region provided by ADNI, comprising the whole cerebellum, brain stem and eroded white matter. Subjects who had at least two scans with zero change in baseline corrected SUVR were discarded. Additionally, a single subject was removed from the ${\text{A}}^{-}{\text{T}}^{-}$ ADNI cohort due to suspected non-AD related tau pathology.

We also use data from the Swedish BioFINDER-2 study (NCT03174938), which uses the RO948 tau PET radiotracer. All participants were recruited at Skåne University Hospital and the Hospital of Ängelholm, Sweden and the cohort covers the full spectrum of AD, ranging from cognitively normal individuals, patients with mild cognitive impairment (MCI) and with dementia. All details about the cohort have been described previously [63]. Amyloid status was determined by amyloid-PET (flutemetamol) with the pons used as the reference region, based on a previously established cut-off from Gaussian mixture modelling as detailed in [60], with positivity requiring flutemetamol SUVR >1.03 in a cortical composite region. The tau data are analysed using the analysis pipeline detailed in [63]. Briefly, SUVR images were generated using the inferior cerebellum as a reference region, and average SUVR was extracted for regions in the DK atlas. Four subjects in the BF2 ${\text{A}}^{+}{\text{T}}^{-}$ group are removed due to high off-target binding in the skull/meninges or MRI registration problems. In both cohorts we select only subjects who have at least three tau PET scans to allow for inference on the time-series model. ADNI and BF2 tau PET data are summarised in Table 3.

**Table 3:** Demographics for ADNI and BF2 cohorts. Aβ mean and standard deviations are provided in centiloids.

	Group	N Sub.	Age	Female	Edu.	CN	MCI	AD	Aβ Mean	Aβ s.d.
ADNI	${\text{A}}^{+}{\text{T}}^{+}$	57	72.61	0.54	16.37	0.28	0.53	0.19	82.26	34.01
	${\text{A}}^{+}{\text{T}}^{-}$	37	76.23	0.49	16.49	0.70	0.32	0.03	49.15	38.42
	${\text{A}}^{-}{\text{T}}^{-}$	52	71.37	0.53	16.48	0.54	0.44	0.02	0.55	13.67
BF2	${\text{A}}^{+}{\text{T}}^{+}$	54	73.9	0.65	11.6	0.2	0.43	0.37	70.68	30.59
	${\text{A}}^{+}{\text{T}}^{-}$	18	72.1	0.5	13.2	0.61	0.31	0.08	24.56	31.89
	${\text{A}}^{-}{\text{T}}^{-}$	53	66.9	0.5	12.8	0.81	0.19	0.0	-7.31	5.98

We perform inference over three groups: ${\text{A}}^{-}{\text{T}}^{-}$, ${\text{A}}^{+}{\text{T}}^{-}$, ${\text{A}}^{+}{\text{T}}^{+}$ We distinguish between ${\text{T}}^{-}$ and *T*^ + ^ using a tau PET SUVR cut-off for two composite regions, as detailed in [34]. There are two cut-offs, one for determining tau positivity in the medial temporal lobe (MTL, defined as the mean of the bilateral entorhinal and amygdala), and another for neocortical positivity (defined as the middle temporal and inferior temporal gyri). The thresholds for the composite regions are based on regional Gaussian mixture models, as previously described [24]. For each composite, we average the SUVR values from the constituent regions and fit a two component Gaussian mixture model. The threshold for the region is then set to the SUVR at which there is a 50% chance of being *T*^ + ^. For ADNI, the thresholds are 1.375 and 1.395 and for BF2 they are 1.248 and 1.451 for the MTL and cortical composites, respectively. We define a subject as being *T*^ + ^ if their last scan is supra-threshold in either the MTL or cortical tau PET SUVR and ${\text{T}}^{-}$ if the SUVR value is below both SUVR thresholds.

### Structural connectome modelling

We use the structural connectome to model the transport of tau between brain regions. To generate structural connectomes, we use diffusion weighted MRI images of 150 healthy individuals from the Human Connectome Project (HCP) [64, 65]. From these data, connectomes are derived using the probabilistic tractography algorithm probtrackx [66], available in FSL, using 10000 samples per voxel, randomly sampled from a sphere around the voxel centre. The number of streamlines between each of *R* regions in the DK atlas are summarised as an adjacency matrix, **A**, that defines our connectome graph, *G*. To model transport of tau between regions, we use the graph Laplacian of *G*, given by:

𝐋=𝐃−𝐀, (3)

where **D** is the degree matrix, 𝐃=diag(𝐀·1). To ensure a transport process respects mass conservation across regions of varying volumes, we weight the graph Laplacian by regional volumes,

$$ℒ={𝐕}^{-1}𝐋$$ (4)

where $𝐕=\text{diag}\left(𝐯/v_{r}\right)$, and $𝐯=(v_{1},v_{2},\cdots v_{R}$) is a vector of regional volumes and $v_{r}$ is a reference region. We model three groups of subjects, ${\text{A}}^{-}{\text{T}}^{-}$, ${\text{A}}^{+}{\text{T}}^{-}$ and ${\text{A}}^{+}{\text{T}}^{+}$, to reflect changes in volume across the disease timeline and variation in individual brain volumes, we define **v** and $v_{r}$ on a group and individual level, respectively. For a given group with *N* subjects, we define the normalised volume of a region $v_{i}$ as

$$v_{i}=\frac{1}{N}\sum_{n}^{N}\frac{v_{i}^{n}}{v_{r}^{n}},$$ (5)

taking $v_{i}^{n}$ as the initial volume of the *i*th region and *n*th subject and $v_{r}^{n}$ is as the maximum initial regional volume for the *n*th subject. Then **v** is the average normalised volume per subject in a cohort.

The graph Laplacian is used in the next section to derive models of tau propagation on the brain network.

### Local model of tau proliferation

We start with a coupled model of healthy and toxic protein, the heterodimer model, from which we aim to derive a simplified model of toxic protein dynamics that includes regional information. The heterodimer model on a network is:

$$\frac{dp_{i}}{\text{d}t}=-\rho \sum_{j=1}^{R}{ℒ}_{ij}p_{j}+k_{0}\;\;-k_{1}p_{i}-k_{12}p_{i}{\tilde{p}}_{i},\;i=1,\ldots ,R,$$ (6a)

$$\frac{d{\tilde{p}}_{i}}{\text{d}t}=-\rho \sum_{j=1}^{R}{ℒ}_{ij}{\tilde{p}}_{j}\;\;-{\tilde{k}}_{1}{\tilde{p}}_{i}+k_{12}p_{i}{\tilde{p}}_{i},\;i=1,\ldots ,R,$$ (6b)

where *p*_*i*_ , $\tilde{p}$ are, respectively, the healthy and toxic protein concentration at node *i*, *k*_0_ is the natural production rate of healthy protein, *k*_1_ and ${\tilde{k}}_{1}$ are the clearance rates of healthy and toxic proteins, respectively, and *k*_12_ is the rate of conversion from healthy proteins into toxic proteins [36]. To simplify the heterodimer model, we can follow a similar procedure to that presented in [36], by linearising around a healthy state. Assuming an homogenous state with ${\tilde{p}}_{i}\ll p_{i}$, implies $\frac{dp_{i}}{dt}=0$ and $-\sum_{j}^{R}{ℒ}_{ij}p_{j}=0$ for i=1⋯R. Then, linearising around $\tilde{p}=0$, we have


$$p_{i}({\tilde{p}}_{i})\approx \frac{k_{0}}{k_{1}}\left(1-\frac{k_{12}}{k_{1}}{\tilde{p}}_{i}\right).$$

Substituting this expression for *p*_*i*_ into Eq (6*b*) we obtain,

$$\frac{d{\tilde{p}}_{i}}{\text{d}t}=-\rho \sum_{j=1}^{R}{ℒ}_{ij}{\tilde{p}}_{j}+\alpha \tilde{p_{i}}-\beta {\tilde{p_{i}}}^{2},\;i=1,\ldots ,R,$$ (7)

where

$$\beta =\frac{k_{0}}{k_{1}}k_{12}-{\tilde{k}}_{1}\;\text{and}\;\alpha =\frac{k_{0}k_{12}^{2}}{k_{1}^{2}}.$$ (8)

From here we derive a model for tau PET SUVR, $s_{i}=s_{i}(t)$ for i=1,…,R, with regional carrying capacities and baseline values to model with the requirement that at node *i*, the healthy state corresponds to a baseline value of $s_{i}=s_{0,i}$ at *t* = 0 and the fully toxic state has asymptotic value $s_{i}=s_{\infty ,i}$ as t→±∞. To accommodate regionally varying carrying capacities and a regionally uniform production rate, we assume regionally varying clearance, ${\tilde{k}}_{1}\to {\tilde{k}}_{1,i}$ for i=1,…,R, making $\beta \to \beta_{i}$ regionally dependent. With this assumption, we can introduce the *local FKPP* model

$$\frac{ds_{i}}{\text{d}t}=-\rho \sum_{j=1}^{R}{ℒ}_{ij}\left(s_{j}-s_{0,j}\right)+\alpha \left(s_{i}-s_{0,i}\right)\left[\left(s_{\infty ,i}-s_{0,i}\right)-\left(s_{i}-s_{0,i}\right)\right],\;i=1,\ldots ,R,$$ (9)

where $\left(s_{i}-s_{0,i}\right)$ represents a shift to regionally dependent non-zero baseline SUVR values and $s_{\infty ,i}=\beta_{i}\alpha^{-1}+s_{0,i}$ is the region carrying capacity at node *i*.

Then, the *logistic model* is then simply obtained by taking ρ=0:

$$\frac{ds_{i}}{\text{d}t}=\alpha \left(s_{i}-s_{0,i}\right)\left[\left(s_{\infty ,i}-s_{0,i}\right)-\left(s_{i}-s_{0,i}\right)\right],\;i=1,\ldots ,R,$$ (10)

where at each node the variable *s*_*i*_ connects asymptotically, for t→±∞, the healthy state *s*_0,*i*_ to the toxic state $s_{\infty ,i}$ (which implies there is no mechanism for propagation from node to node in this model). The diffusion model assumes α=0 in Eq (9):

$$\frac{ds_{i}}{\text{d}t}=-\rho \sum_{j=1}^{R}{ℒ}_{ij}\left(s_{j}-s_{0,j}\right),\;i=1,\ldots ,R,$$ (11)

where there is no mechanism for production. The *global FKPP* model is taken by assuming regionally homogeneous baseline values and carrying capacities across all nodes:

$$\frac{ds_{i}}{\text{d}t}=-\rho \sum_{j=1}^{R}{ℒ}_{ij}\left(s_{i}-s_{0}\right)+\alpha (s_{i}-s_{0})\left[(s_{\infty }-s_{0})-(s_{i}-s_{0})\right],\;i=1,\ldots ,R.$$ (12)

A method for determining the values of $s_{\infty }$, $s_{0}$, $s_{\infty }$, and *s*_0_ are provided in the next section.

### Estimating fixed model parameters

To estimate the fixed parameters for ${𝐬}_{0}$ and ${𝐬}_{\infty }$, we fit a two component Gaussian mixture model to population level data of regional SUVR. For regions in which a reliable measure of tau SUVR can be obtained, we expect to see two separate distributions, a ${\text{T}}^{-}$ distribution capturing the expected tau load in a given region, and a *T*^ + ^ distribution describing the pathological tau load [24]. Using the fitted Gaussian mixture models, we approximate *s*_0,*i*_ as the mean of the ${\text{T}}^{-}$ distributions for the *i*-th region and $s_{\infty ,i}$ as the 99-th percentile of the *T*^ + ^ distributions for the *i*-th. These parameters are used to simulate Eqs (9) to (11). To simulate from Eq (12) we take $s_{\infty }$ as $\max(s_{\infty })$ and *s*_0_ as $\min(s_{0})$. For subcortical regions it is not possible to obtain reliable tau PET signal due to off-target binding [67, 68] and we therefore exclude these regions from our model, leaving a total of 72 regions. For ADNI, we use the multi-tau cohort of AV1451 PET data, detailed in [24]. For BF2 data, we use all available RO948 PET scans. For all regions used, including the bilateral hippocampus and amygdala, a Gaussian mixture with two components provided a better fit to the data than a single component data, compared using the AIC score.

### Probabilistic model

For each of the three groups, ${\text{A}}^{+}{\text{T}}^{+}$, ${\text{A}}^{+}{\text{T}}^{-}$, ${\text{A}}^{-}{\text{T}}^{-}$, we use a hierarchical model, factoring over patients and scans. In each group there are *N* subjects, each of whom have *T*_*n*_ scans, for n=1⋯N subjects, summarised over *R* regions, (R=72). The observations times, i.e. scan dates, are denoted by $𝐭=t_{j}^{n}$ for $j=1\cdots T_{n}$, n=1⋯N. We denote the full data set for a group as **Y** and individual subject data as $Y_{ij}^{n}$, corresponding to the *n*th subject, at scan *j* and region *i*. For a single subject, we have the following data generating function:

$${𝐘}^{n}=𝐟({𝐲}_{0}^{n},\theta^{n},{𝐭}^{n})+\epsilon .$$ (13)

where ${𝐘}^{n}$ is the individual data for *R* regions and *T*_*n*_ time points, with initial conditions ${𝐲}_{0}$, model parameters θ, and observations times **t**. The data are generated by a dynamical systems, **f**, with observation error ϵ. To derive a likelihood function from Eq (13), we assume the observations errors are independently and identically distributed and sampled from a Gaussian distribution with standard deviation σ. The data generating distribution for a single observation from a subject is then:

$$\epsilon \sim 𝒩(0,\sigma^{2})$$ (14)

$${𝐘}^{n}\sim 𝒩(𝐟({𝐲}_{0}^{n},\theta ,{𝐭}^{n}),\sigma^{2}𝐈)$$ (15)

To extend this to a hierarchical population model, we define random variables, $\Theta =\{(\rho_{i},\alpha_{i}){\}}_{i=1}^{N}$, encoding subject specific model parameters and hierarchical population parameters, $\Omega =\{\rho_{\mu },\rho_{\sigma },\alpha_{\mu },\alpha_{\sigma }\}$, upon which each $\Theta_{i}$ depends. For each subject, we assume fixed initial conditions, ${𝐲}_{0}^{n}$, and observations times, ${𝐭}^{n}$, taken as the first tau PET scan and scan dates respectively. Then the likelihood function for the n-th subject under the hierarchical model is:

$$p({𝐘}^{n},\Theta_{n}|\Omega ,\sigma ,{𝐲}_{0}^{n},{𝐭}^{n})=\prod_{j}^{T_{n}}\prod_{i}^{R}p(Y_{ij}^{n}|\Theta_{n},\sigma ,{𝐲}_{0}^{n},t_{j}^{n})\;p(\Theta_{n}|\Omega )$$ (16)

where the first term inside the product on the right hand side is the contribution of the subject level model and the second term is the hierarchical model. Then the posterior for all subjects, hierarchical parameters, subject specific parameters and observation noise is:

$$p(\Theta ,\Omega ,\sigma |𝐘,𝐭,{𝐲}_{0})\propto \prod_{n}^{N}p({𝐘}^{n},\Theta_{n}|\Omega ,\sigma ,{𝐲}_{0}^{n},{𝐭}^{n})\;p(\Omega ,\sigma ).$$ (17)

### Inference algorithm

We run inference for each patient group separately using a Hamiltonian Monte Carlo No-U-Turn Sampler (NUTS) to sample from the group posterior distribution. We use the same priors across patient groups, provided in Table 4. We use weakly informative priors based on scales at which we expect to observe parameter values and ensure the transport parameter is positive. The NUTS sampler is initialised with a unit diagonal Euclidean metric and a target acceptance ratio of 0.8. For each patient group, we collected four chains each with 2000 samples. All chains showed good convergence (measured by $0.99<\hat{r}<1.01$) with no post warm-up numerical errors associated with the NUTS sampler.

**Table 4:** Prior distributions for hierarchical model parameters.

Parameter	Prior	Support
$\rho_{\mu }$	Lognormal(0,1)	[0,∞]
$\rho_{\sigma }$	Lognormal(0,1)	[0,∞]
$\alpha_{\mu }$	𝒩(0,1)	[∞,∞]
$\alpha_{\sigma }$	Lognormal(0,1)	[0,∞]
$\rho_{i}$	$𝒩(\rho_{\mu },\rho_{\sigma })$	[0,∞]
$\alpha_{i}$	$𝒩(\alpha_{\mu },\alpha_{\sigma })$	[−∞,∞]
σ	Lognormal(0,1)	[0,∞]

### Model assessment

In Table 1 use two metrics to compare a family of models, the Bayesian information criteria (BIC) and the expected log predictive density (ELPD). The BIC is used to compare the in-sample accuracy of each of the model’s fit to the data using a NUTS sampler, and is given by:

BIC=klog(n)−2logL, (18)

where

$$L=p(𝐘,\Theta^{⋆}|\Omega^{⋆},\sigma^{⋆},{𝐲}_{0},𝐭)$$ (19)

is the total log-likelihood of all data, 𝐘, over subjects, scans and regions, calculated using the parameters $\Theta^{⋆}$, $\Omega^{⋆}$ and $\sigma^{⋆}$. Parameters are chosen from the posterior samples collected during inference as those that maximise the log-likelihood. *k* is the number combined number of parameters between $\Theta^{⋆}$, $\Omega^{⋆}$ and $\sigma^{⋆}$ (*k* = 119 for the local and global FKPP models; *k* = 60 for the diffusion and logistic models); *n* = 13536 is the total number of observations.

The ELPD is used for estimating the out-of-sample predictive accuracy and is adapted from [69]. To do this, we use ${\text{A}}^{+}{\text{T}}^{+}$ subjects who have more than three tau PET scans (N = 10), using only the first three scans for training and remaining scans to measure predictive accuracy. For our model, the ELPD is then calculated as:

$$\text{ELPD}=\sum_{n}^{N}\left[\log\left(\frac{1}{S}\sum_{s}^{S}p\left({𝐘}^{n}|\Theta_{s}^{n},{𝐲}_{0}^{n},{𝐭}^{n}\right)\right)\right]$$ (20)

where ${𝐘}^{n}$ are the unobserved data, $\Theta_{s}^{n}=\{(\rho ,\alpha ){\}}_{s=1}^{S}$ are posterior samples of model parameters, ${𝐲}_{0}^{n}$ are subjects initial condition and ${𝐭}^{n}$ are scan dates, each for n=1⋯N subjects.

The final method we use for model assessment is the comparison to shuffled data to examine whether the posterior distributions generated inferred from the true data are due to meaningful tau signal or statistical properties of the data. We perform this test on the ${\text{A}}^{+}{\text{T}}^{+}$ and ${\text{A}}^{+}{\text{T}}^{-}$ groups by random spatial shuffling of the data. The same random permutation are applied to the regional baseline values and carrying capacities. The inference algorithm was then applied to the shuffled dataset and 1000 posterior samples were collected. This process was repeated 10 times for each group. In the ${\text{A}}^{+}{\text{T}}^{+}$ positive group, one chain failed to converge and was discarded.

## Supporting information

S1 FigResidual analysis for in-sample model fit.Regional residual averaged over subjects in the ${\text{A}}^{+}{\text{T}}^{+}$ cohort, showing the regionally averaged difference between the SUVR of final scans and the corresponding prediction using the local FKPP, global FKPP and logistic models. Black dashed line represents the mean error averaged over subjects and regions. Blue solid line highlights zero error(PDF)

S2 FigOut-of-sample fit and posterior predictive plots for the left inferior temporal region.The local FKPP model was iteratively calibrated to a ${\text{A}}^{+}{\text{T}}^{+}$ ADNI cohort with 41 in-sample subjects and 16 test subjects. Three iteration were run where for each iteration an additional scan from the test subjects were included, starting with a single scan. Posterior predictive trajectories for left inferior temporal lobe are shown for each iteration (neglecting observation noise). In the above figure, each panel represents one of the 16 test subjects. Each point represents a data point added for a training iteration; trajectories are colour matched to correspond to the number of longitudinal data points included for training.(PDF)

S3 FigOut-of-sample fit and posterior predictive plots for the left entorhinal cortex.The local FKPP model was iteratively calibrated to a ${\text{A}}^{+}{\text{T}}^{+}$ ADNI cohort with 41 in-sample subjects and 16 test subjects. Three iteration were run where for each iteration an additional scan from the test subjects were included, starting with a single scan. Posterior predictive trajectories for left entorhinal cortex are shown for each iteration. In the above figure, each panel represents each of the 16 test subjects. Each point represents a data point added for training iteration; trajectories are colour matched to correspond to the number of longitudinal data points included for training.(PDF)

S4 FigOut-of-sample fit and posterior predictive plots for the left entorhinal cortex with the addition of observation noise.The local FKPP model was iteratively calibrated to a ${\text{A}}^{+}{\text{T}}^{+}$ ADNI cohort with 41 in-sample subjects and 16 test subjects. Three iteration were run where for each iteration an additional scan from the test subjects were included, starting with a single scan. Posterior predictive trajectories for left entorhinal cortex are shown for each iteration. In the above figure, each panel represents each of the 16 test subjects. Each point represents a data point added for training iteration; trajectories are colour matched to correspond to the number of longitudinal data points included for training.(PDF)

S5 FigPopulation and Individual level posterior distributions from ADNI.Population-level posterior distributions (left) and individual level distributions (right) for the transport (top) and production (bottom) parameters inferred from the ${\text{A}}^{+}{\text{T}}^{+}$, ${\text{A}}^{+}{\text{T}}^{-}$ and ${\text{A}}^{-}{\text{T}}^{-}$ groups in ADNI. Data underlying this figure can be found at https://doi.org/10.5281/zenodo.15389493.(PDF)

S6 FigPopulation and Individual level posterior distributions from BF-2.Population-level posterior distributions (left) and individual level distributions (right) for the transport (top) and production (bottom) parameters inferred from the ${\text{A}}^{+}{\text{T}}^{+}$, ${\text{A}}^{+}{\text{T}}^{-}$ and ${\text{A}}^{-}{\text{T}}^{-}$ groups in BF-2. Data underlying this figure can be found at https://doi.org/10.5281/zenodo.15389493.(PDF)

S7 FigRegional Change in SUVR vs Cortical Thickness.The first three panels show the correlation between change in cortical thickness and change in SUVR for the ${\text{A}}^{-}{\text{T}}^{-}$, ${\text{A}}^{+}{\text{T}}^{-}$, ${\text{A}}^{+}{\text{T}}^{+}$ groups. Each point represents a cortical region averaged over individuals. Cortical thickness data was obtained as preprocessed tabular data from ADNI and time-matched with individual PET scans. We exclude the bilateral entorhinal cortex as an outlier since it displays high atrophy despite the absence of AD-pathology, leaving *R* = 66 regions. Note that the global production rate will be dominated by the average change in SUVR. There is a positive correlation between longitudinal change in cortical thickness vs change in SUVR for the ${\text{A}}^{-}{\text{T}}^{-}$ group, suggesting the negative production rate observed in the ${\text{A}}^{-}{\text{T}}^{-}$ group is a result of decreasing SUVR resulting from atrophy. As expected, the correlation between change in thickness vs change in SUVR becomes negative for ${\text{A}}^{+}{\text{T}}^{-}$ and ${\text{A}}^{+}{\text{T}}^{+}$ groups, indicating that tau progression outpaces atrophy. The final panel shows the correlation between the α parameter and the change in thickness for the ${\text{A}}^{-}{\text{T}}^{-}$ group.(PDF)

S1 Text Effects Of Data Processing Choices On Longitudinal Modelling. (PDF)
