# Supplementary figures and images for: Personalised regional modelling predicts tau progression in the human brain
Source: PLoS Biol. 2025 Jul 21;23(7):e3003241. doi: 10.1371/journal.pbio.3003241 (PMC12303394; doi:10.1371/journal.pbio.3003241)

**Local FKPP**

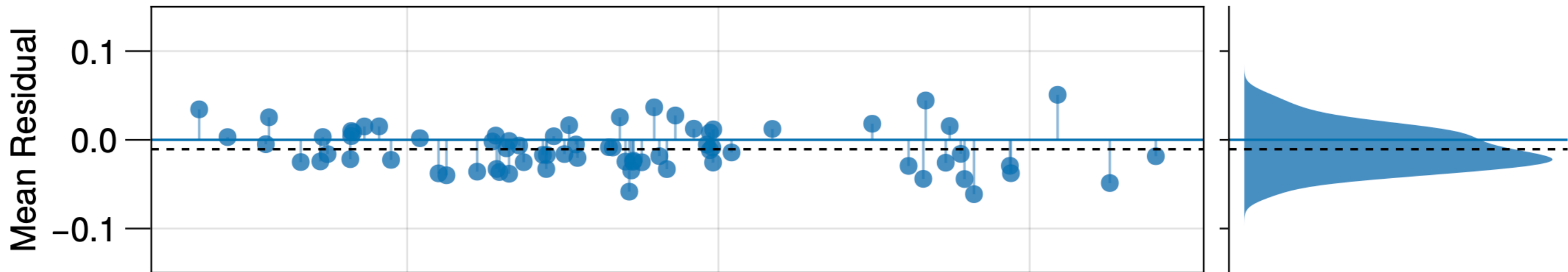

**Global FKPP**

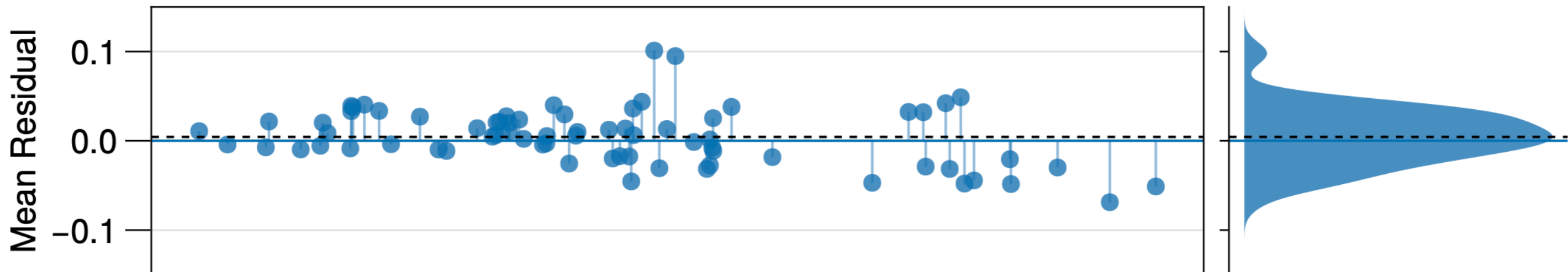

**Logistic**

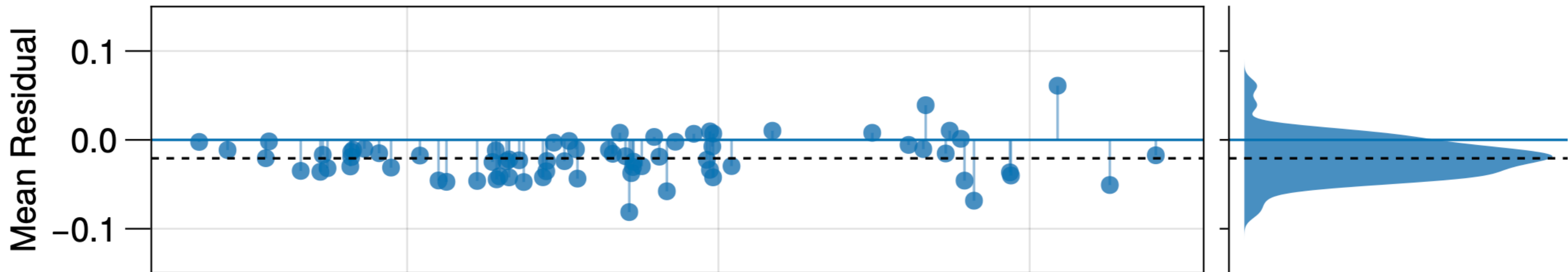

Supplement: S1 Fig — Regional residual averaged over subjects in the A+T+ cohort, showing the regionally averaged difference between the SUVR of final scans and the corresponding prediction using the local FKPP, global FKPP and logistic models. Black dashed line represents the mean error averaged over subjects and regions. Blue solid line highlights zero error (PDF) [file pbio.3003241.s001.pdf]

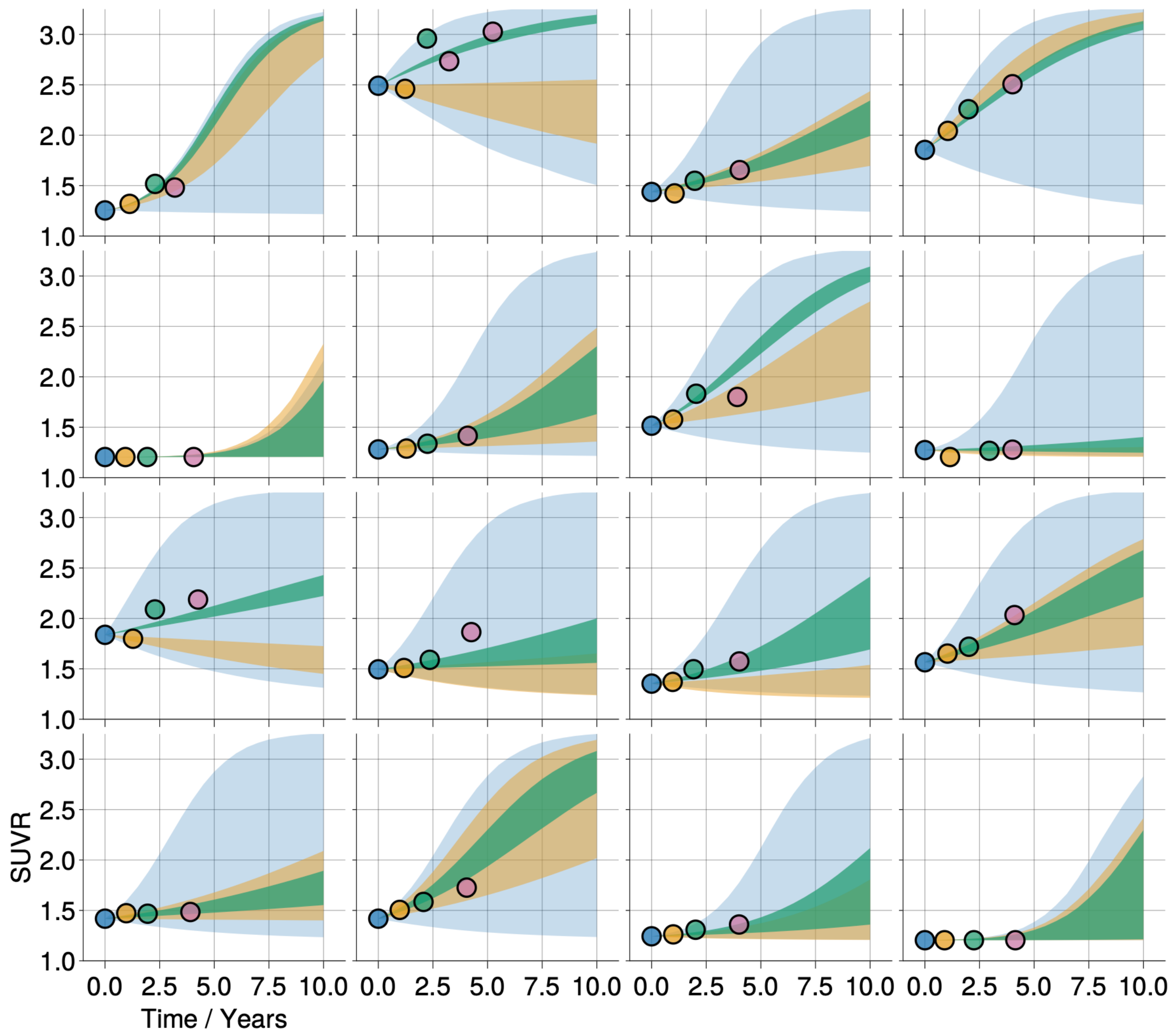

Supplement: S2 Fig — The local FKPP model was iteratively calibrated to a A+T+ ADNI cohort with 41 in-sample subjects and 16 test subjects. Three iteration were run where for each iteration an additional scan from the test subjects were included, starting with a single scan. Posterior predictive trajectories for left inferior temporal lobe are shown for each iteration (neglecting observation noise). In the above figure, each panel represents one of the 16 test subjects. Each point represents a data point added for a training iteration; trajectories are colour matched to correspond to the number of longitudinal data points included for training. (PDF) [file pbio.3003241.s002.pdf]

## Transport

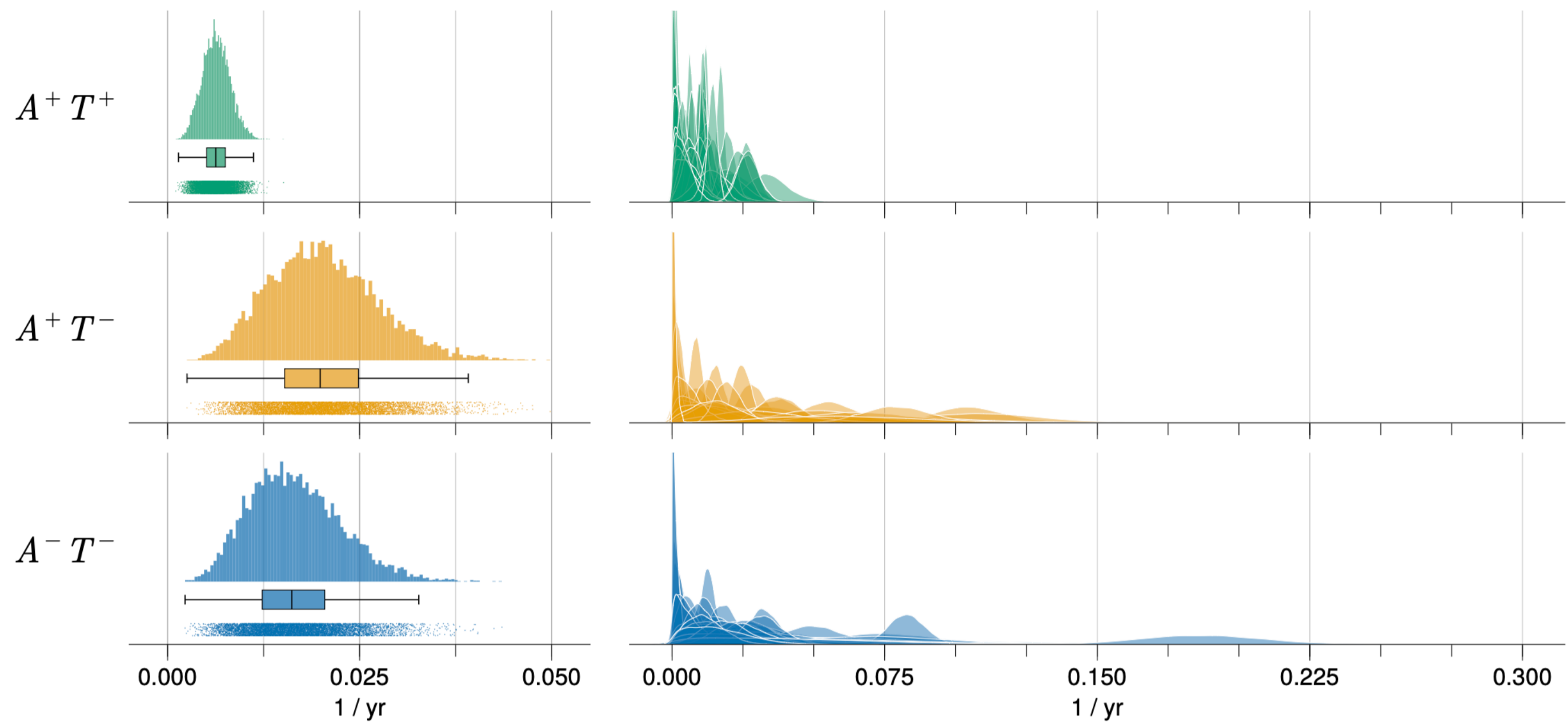

## Production

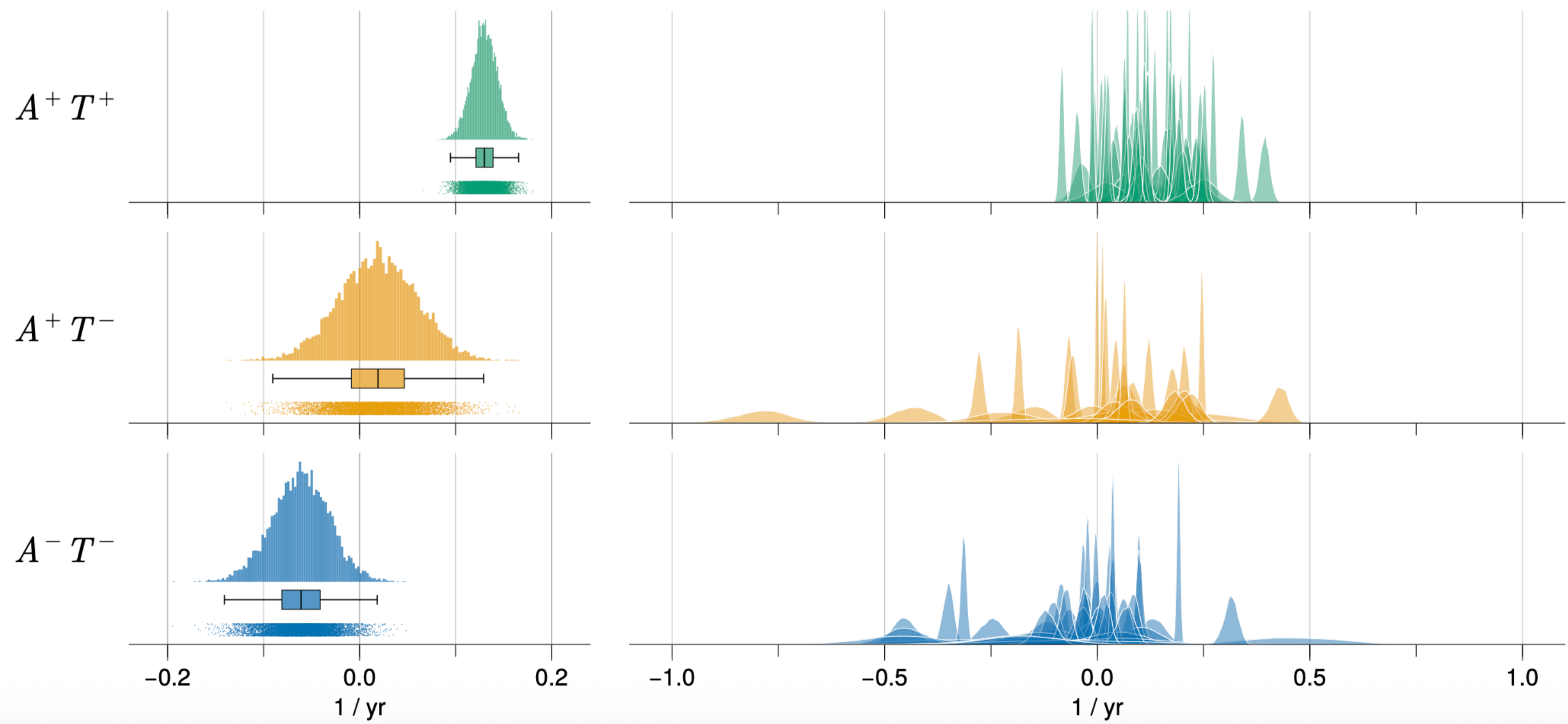

Supplement: S5 Fig — Population-level posterior distributions (left) and individual level distributions (right) for the transport (top) and production (bottom) parameters inferred from the A+T+, A+T− and A−T− groups in ADNI. Data underlying this figure can be found at https://doi.org/10.5281/zenodo.15389493. (PDF) [file pbio.3003241.s005.pdf]

# Transport

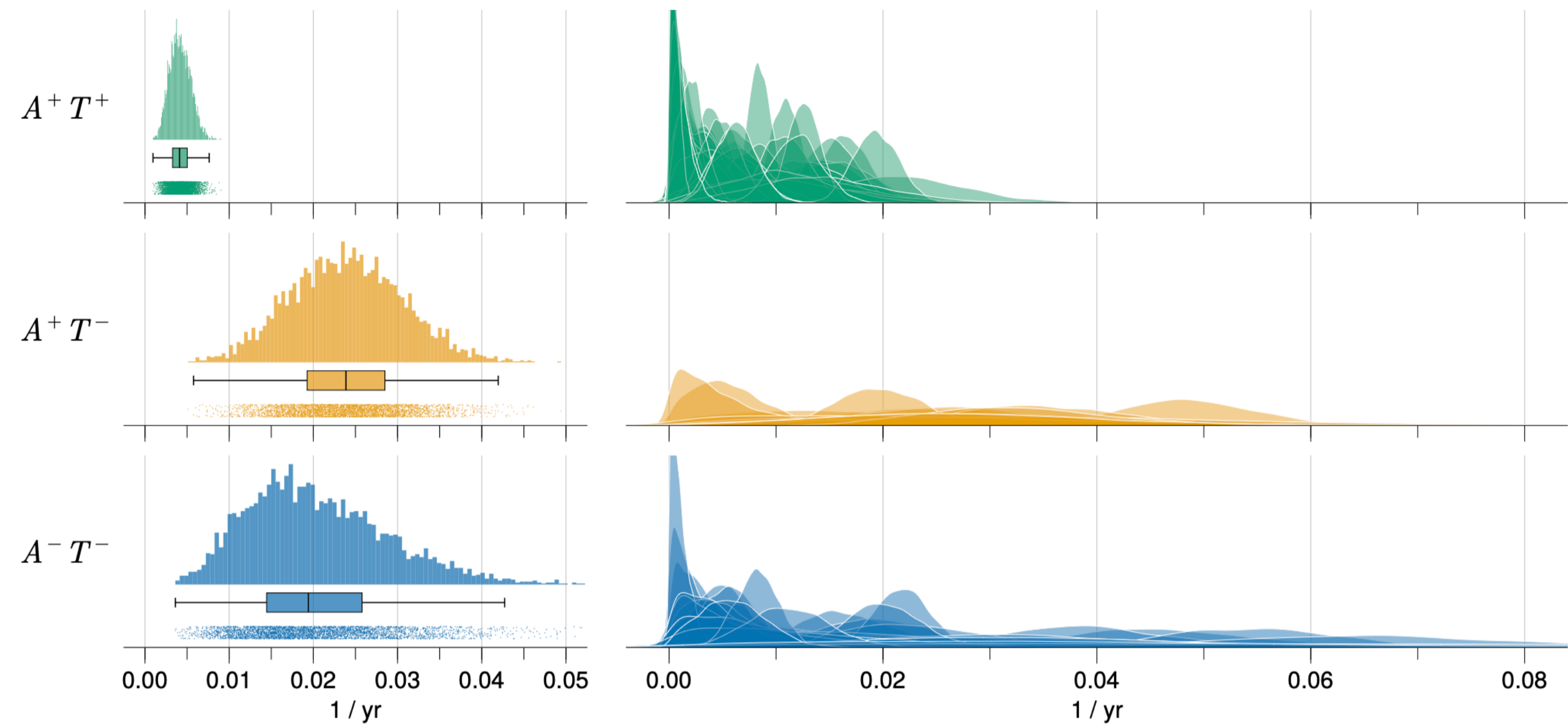

# Production

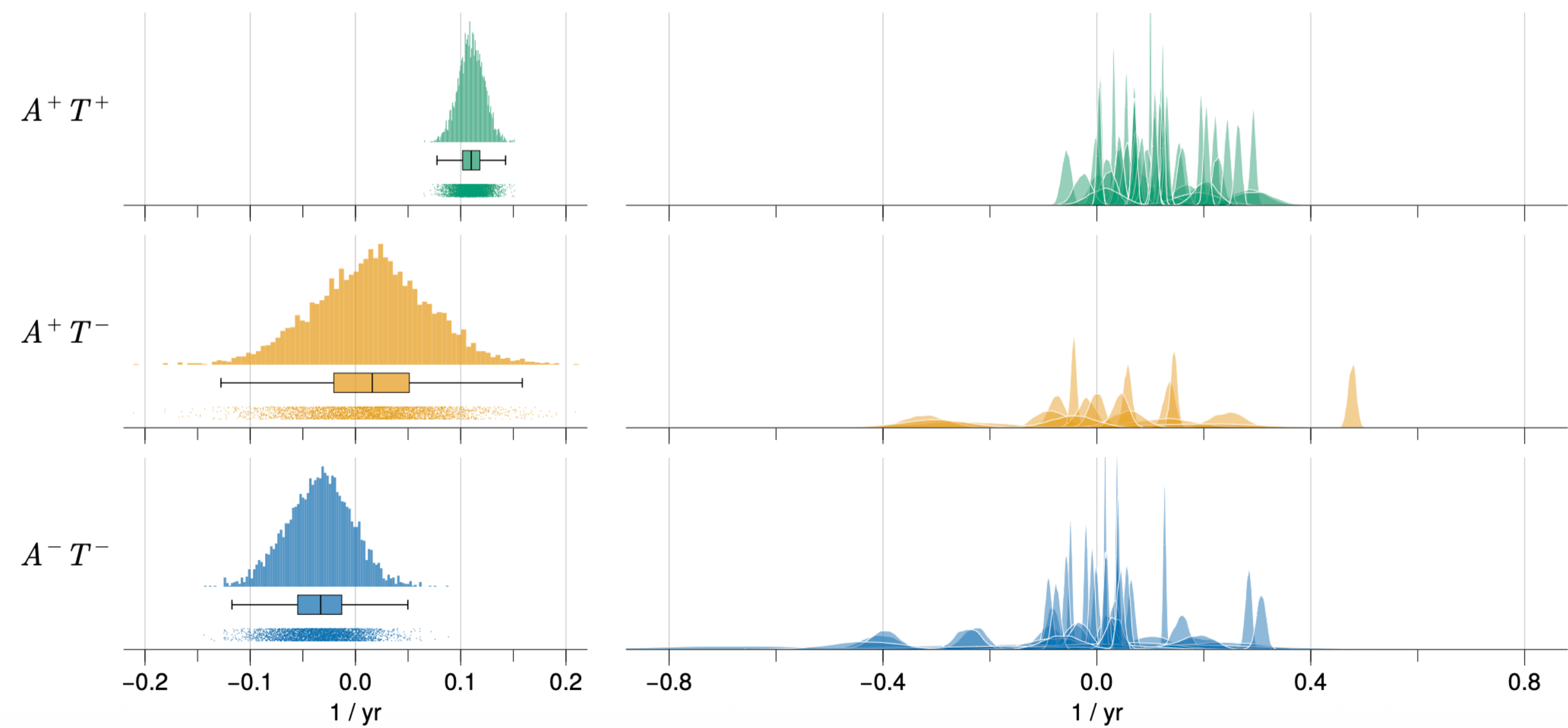

Supplement: S6 Fig — Population-level posterior distributions (left) and individual level distributions (right) for the transport (top) and production (bottom) parameters inferred from the A+T+, A+T− and A−T− groups in BF-2. Data underlying this figure can be found at https://doi.org/10.5281/zenodo.15389493. (PDF) [file pbio.3003241.s006.pdf]

$A^- T^-$ 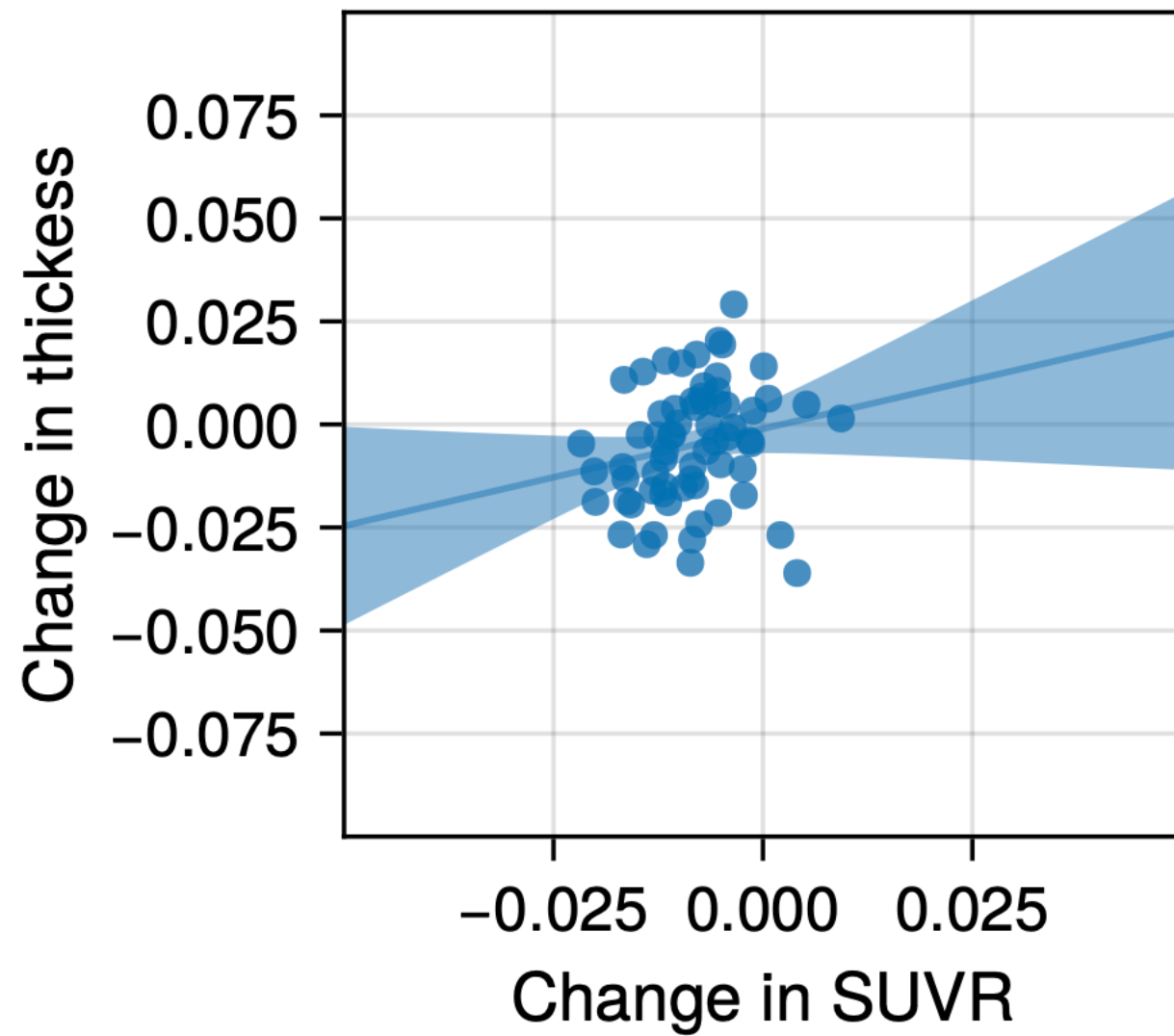 $A^+ T^-$ 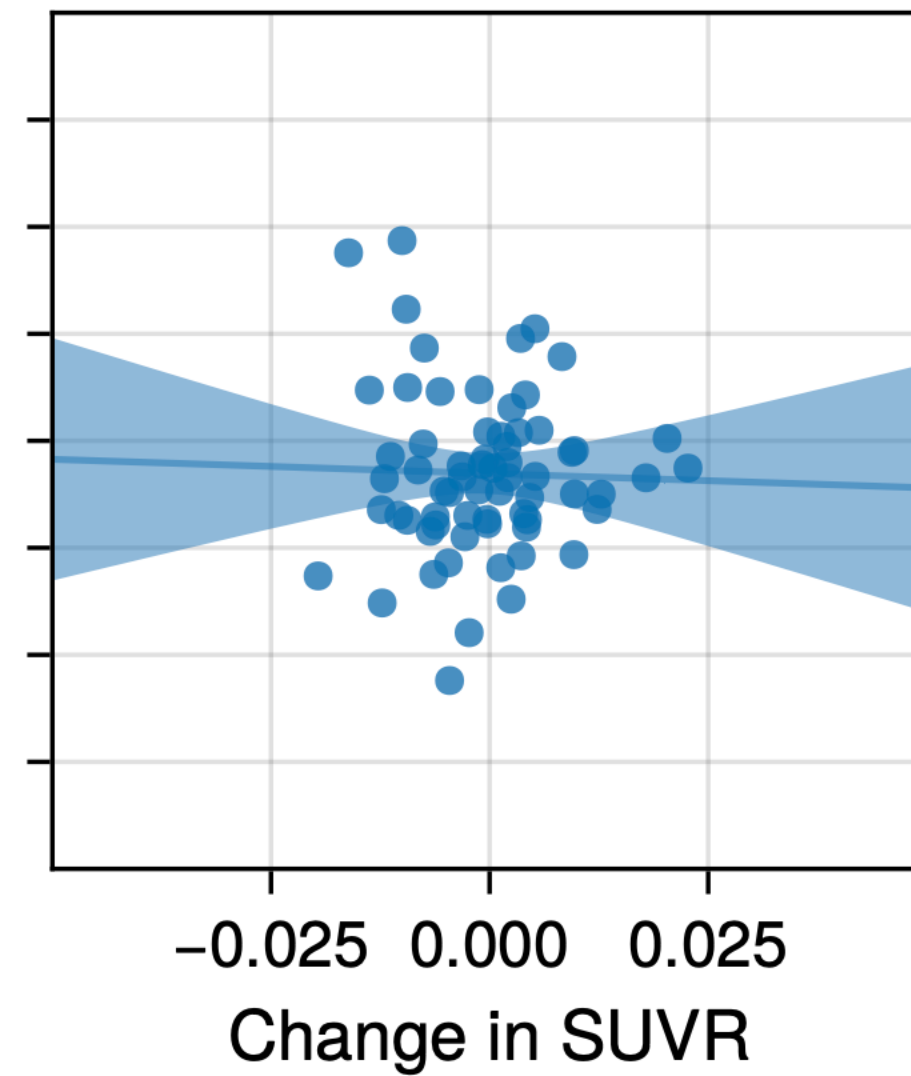 $A^+ T^+$ 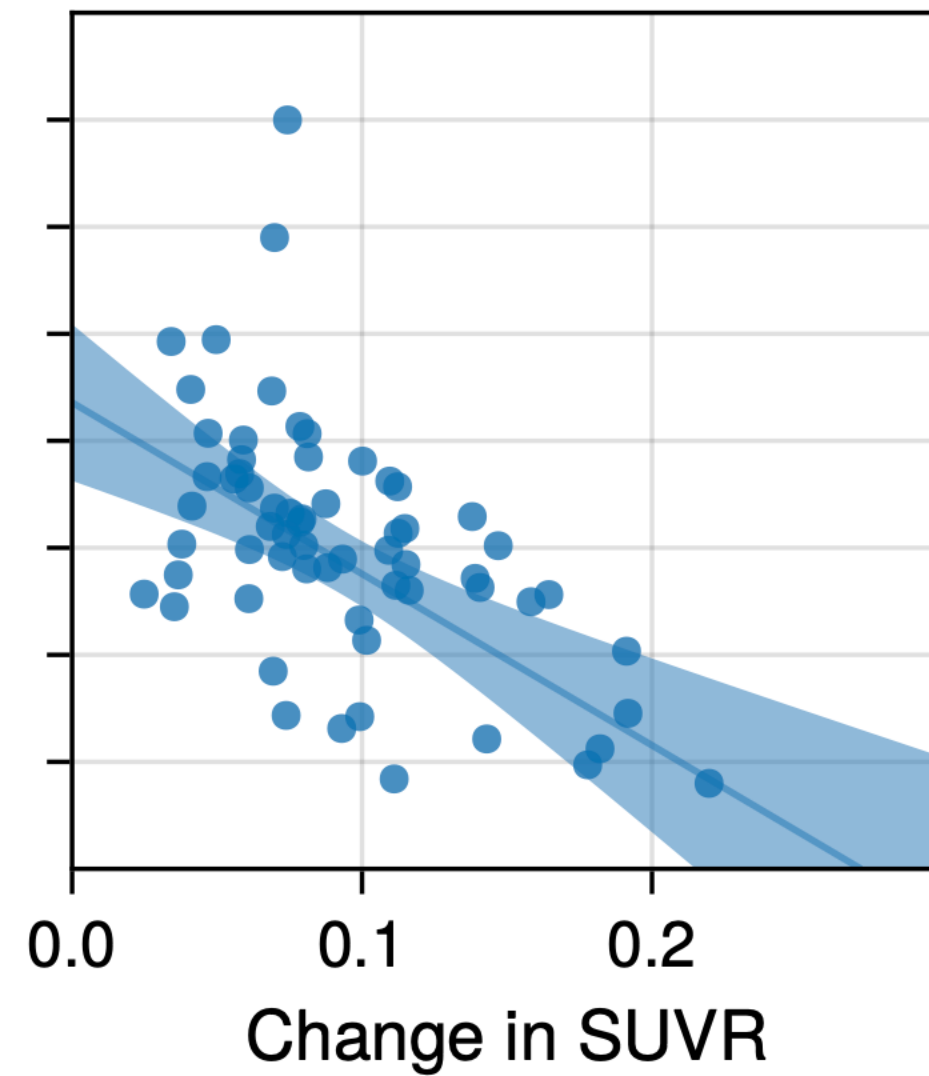 $A^- T^-$ 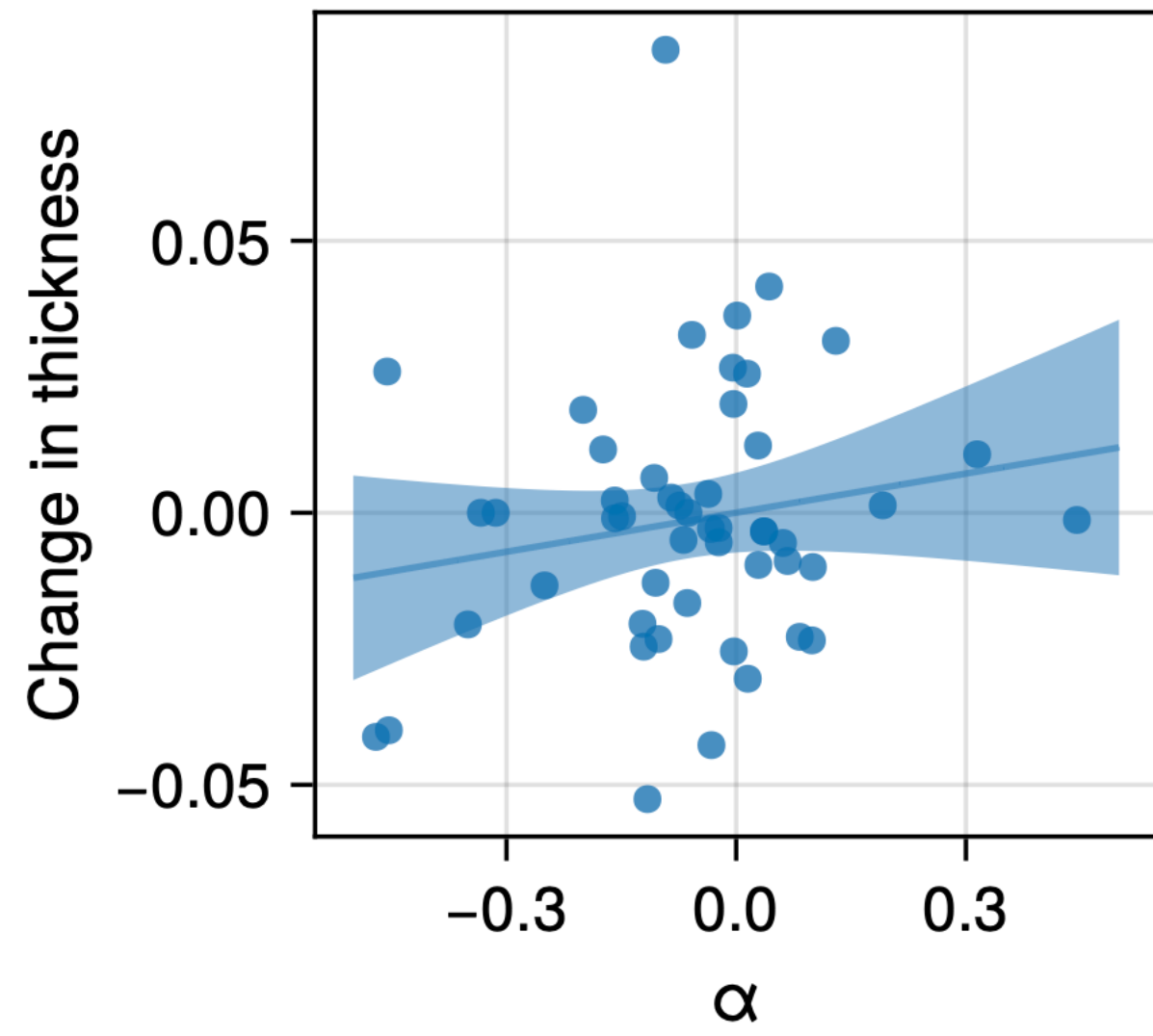

Supplement: S7 Fig — The first three panels show the correlation between change in cortical thickness and change in SUVR for the A−T−, A+T−, A+T+ groups. Each point represents a cortical region averaged over individuals. Cortical thickness data was obtained as preprocessed tabular data from ADNI and time-matched with individual PET scans. We exclude the bilateral entorhinal cortex as an outlier since it displays high atrophy despite the absence of AD-pathology, leaving R = 66 regions. Note that the global production rate will be dominated by the average change in SUVR. There is a positive correlation between longitudinal change in cortical thickness vs change in SUVR for the A−T− group, suggesting the negative production rate observed in the A−T− group is a result of decreasing SUVR resulting from atrophy. As expected, the correlation between change in thickness vs change in SUVR becomes negative for A+T− and A+T+ groups, indicating that tau progression outpaces atrophy. The final panel shows the correlation between the α parameter and the change in thickness for the A−T− group. (PDF) [file pbio.3003241.s007.pdf]
