# Supplementary material for: Personalised regional modelling predicts tau progression in the human brain
Source: PLoS Biol. 2025 Jul 21;23(7):e3003241. doi: 10.1371/journal.pbio.3003241 (PMC12303394; doi:10.1371/journal.pbio.3003241)
Supplement: S4 Fig — The local FKPP model was iteratively calibrated to a A+T+ ADNI cohort with 41 in-sample subjects and 16 test subjects. Three iteration were run where for each iteration an additional scan from the test subjects were included, starting with a single scan. Posterior predictive trajectories for left entorhinal cortex are shown for each iteration. In the above figure, each panel represents each of the 16 test subjects. Each point represents a data point added for training iteration; trajectories are colour matched to correspond to the number of longitudinal data points included for training. (PDF) [file pbio.3003241.s004.pdf]

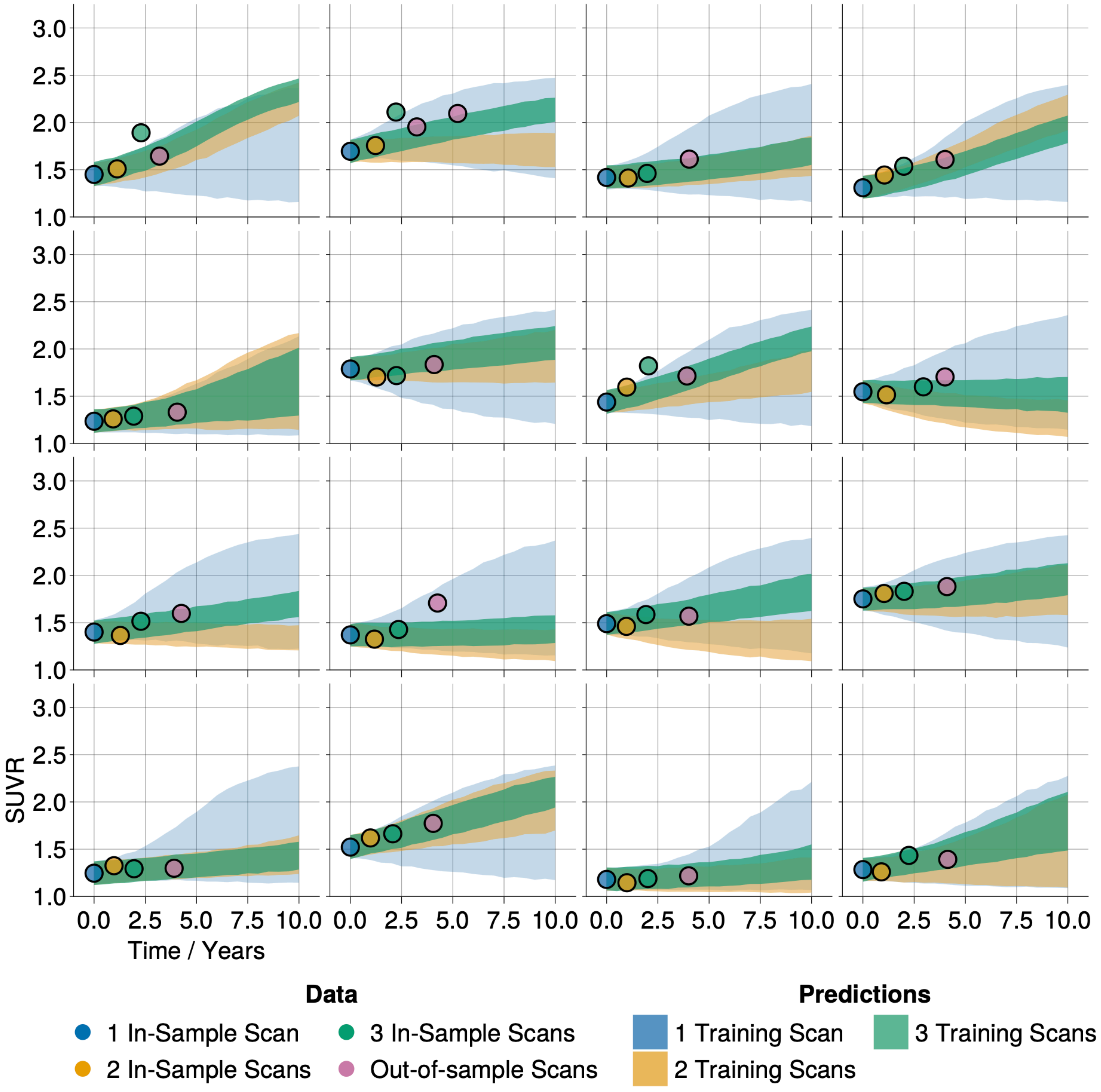

SUVR
Time / Years
Data
1 In-Sample Scan
2 In-Sample Scans
3 In-Sample Scans
Out-of-sample Scans
Predictions
1 Training Scan
2 Training Scans
3 Training Scans
