# Supplementary material for: Personalised regional modelling predicts tau progression in the human brain
Source: PLoS Biol. 2025 Jul 21;23(7):e3003241. doi: 10.1371/journal.pbio.3003241 (PMC12303394; doi:10.1371/journal.pbio.3003241)
Supplement: S1 Text — (PDF) [file pbio.3003241.s008.pdf]

S1 Text

Effects Of Data Processing Choices On Longitudinal Modelling

We reapplied the local FKPP model to ADNI data with two different processing options: (1) partial volume correction and (2) an eroded white matter reference region. The posterior distributions of this analysis are shown in Fig. A.

In an effort to explain difference between the changes in the production rate across AD groups with the eroded white matter, we conducted a further analysis of the longitudinal behaviour of both the eroded white matter and inferior cerebellar reference region. To perform this analysis, we use preprocessed data from ADNI, for which SUVR values are provided using the inferior cerebellum reference for both target regions and alternative reference regions, including the eroded white matter. In the top left panel of Fig. B, we show the longitudinal change in SUVR for regions in the DK atlas, averaged over subjects, using both the inferior cerebellum and the eroded white matter reference regions. The use of the eroded white matter reference region eliminates the negative longitudinal changes observed with the inferior cerebellar reference region for the $A^{+}T^{-}$ and $A^{-}T^{-}$ groups, consistent with the results of model inference in Fig. A. We hypothesized that this is due to atrophy-related effects in the reference region that result in decreasing reference region SUVR. To test this, we conducted further analysis into the longitudinal behaviour of the different reference regions. First, we examine longitudinal volume changes in the reference regions for each subject from baseline scans to final scans, shown in the top right panel of Fig. B. The results show that the volume of the inferior cerebellar reference region is more stable longitudinally than the eroded white matter, which shows greater variability and is more prone to atrophy. Next, we examined longitudinal changes in reference region SUVR from baseline to final scan. In the bottom panel of Fig. B we compare the subject-wise longitudinal change in SUVR of the eroded white matter to longitudinal change in medial temporal SUVR (comprising the bilateral entorhinal cortex and amygdala), where the SUVR is for all regions used is calculated using the inferior cerebellum, as provided by ADNI. We see that there is a strong positive correlation between longitudinal eroded white matter SUVR change and MTL SUVR and that decreasing MTL SUVR is associated with decreases in reference region SUVR. Due to this positive correlation, additional normalisation with the eroded white matter reference region would diminish any longitudinal changes in target regions, such as we see in the top left panel of Fig. B. In sum, these analyses show that the eroded white matter has less stable longitudinal SUVR and volume compared to the inferior cerebellum and its longitudinal SUVR is positively correlated with MTL SUVR, likely resulting in the diminished production effect observed in the inferred posterior distributions in Fig. A.

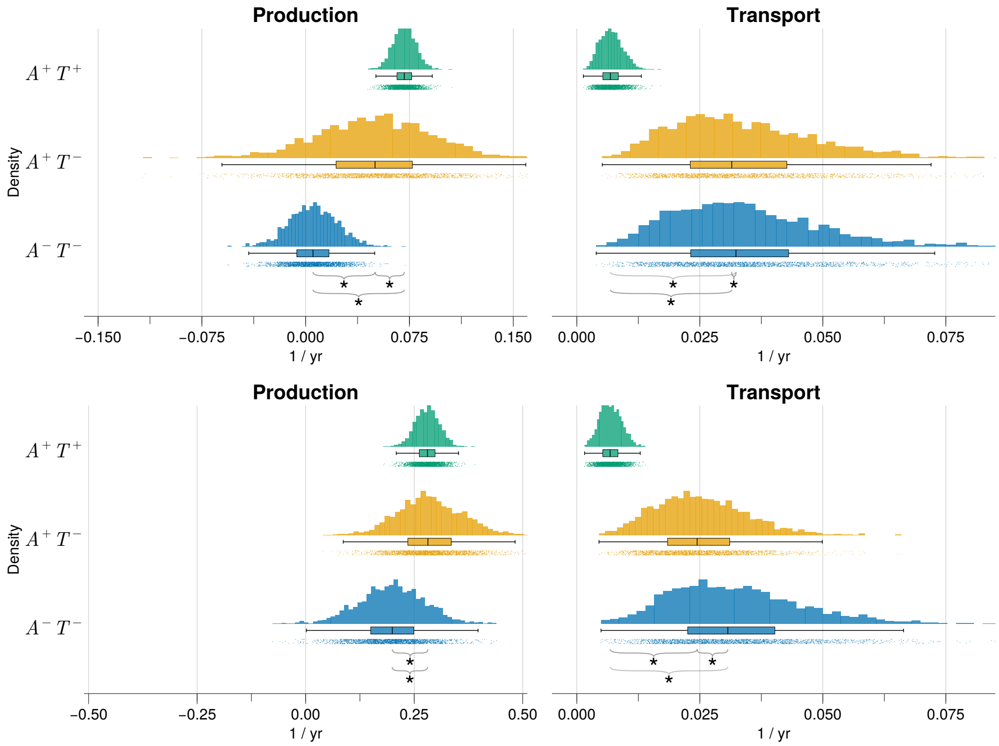

Fig A. Inferred posterior distributions for PVC and EWM ADNI data. (Top) Posterior distributions from $A^{+}T^{+}$, $A^{+}T^{-}$ and $A^{-}T^{-}$ groups for ADNI data processed using an inferior cerebellar reference region and with partial volume correction. (Bottom) Posterior distributions from $A^{+}T^{+}$, $A^{+}T^{-}$ and $A^{-}T^{-}$ groups using ADNI data processed using the eroded white matter reference region. Posterior distributions were obtained using Eq. (17) and an identical inference set up to previous results. We collected 2000 posterior samples from each gropu; there were no divergences and samples shows good effective sample size and $0.99 \leq \hat{r} \leq 1.01$, indicating good convergence. Significance between distributions is given by the Mann-Whitney U test with $P < 0.01$. Data underlying this figure can be found at https://doi.org/10.5281/zenodo.15389493

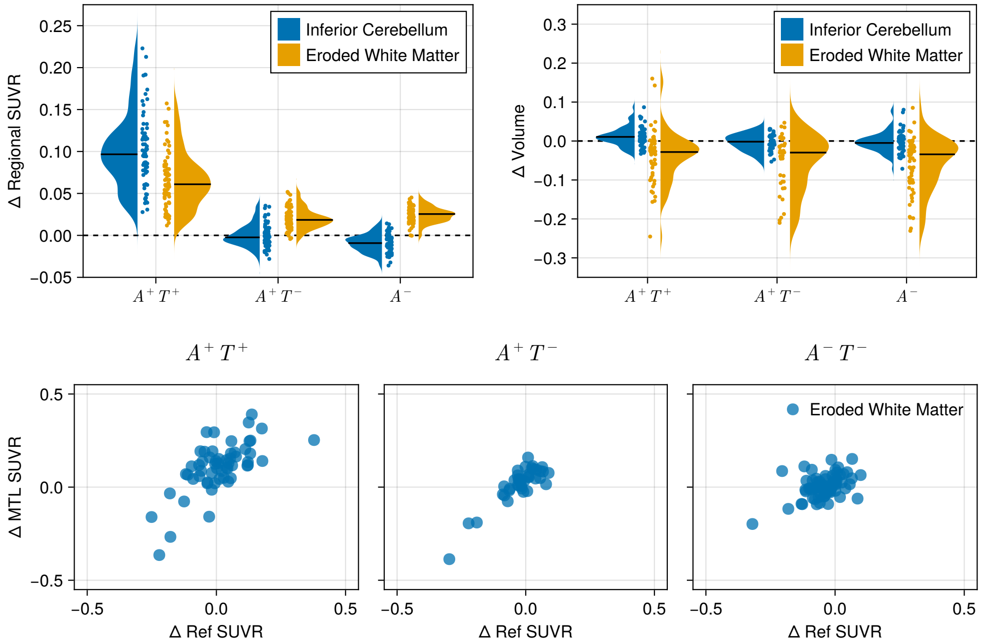

Fig B. Effects of reference region on longitudinal tau PET. (Top Left) Longitudinal change in target regions SUVR from first to last scan in the $A^{+}T^{+}$, $A^{+}T^{-}$ and $A^{-}T^{-}$ ADNI cohorts with inferior cerebellar and eroded white matter reference regions. Each point represents the longitudinal change in SUVR for a region in the DK atlas, averaged over subjects in each cohort. The density of these changes is shown for each group and reference region, with the median change highlighted with a black line through the density. (Top Right) Longitudinal change in reference region volume relative to baseline scan and normalised by total intracranial volume. Each point represents a subject in the ADNI cohort. (Bottom) Longitudinal change in reference region SUVR vs change in MTL (bilateral amygdala and entorhinal cortex) SUVR per subject in the each of the ADNI cohorts. Each point represents a subject in each ADNI cohort. For the eroded white matter and MTL regions, we use the SUVR values based on the inferior cerebellar reference region to highlight the effect of further normalisation using a different reference region, namely the eroded white matter.

Re-Analysis on Schaefer-200 Atlas

We investigate the role of parcellation and connectome on the results presented in Fig. 4. We rerun our analysis using the Schaefer-200 atlas provided by the ENIGMA project [1]. The larger parcellations means individual parcels have more uniform volumes. We expect this to more accurately detect transport dynamics that may be lost averaging signal over large regions, such as those in the DK atlas. We rerun this analysis on the BF-2 dataset, using the same subjects cohorts used as in Fig. 4. First, we estimate regional parameter vectors using Gaussian mixture modelling, as described in the Methods section. Excluding regions that are not best captured by a two-component Gaussian mixture model there are $R = 198$ regions in total. We re-apply the model Eq. (17) to the $A^{+}T^{+}$, $A^{+}T^{-}$ and $A^{-}T^{-}$ BF-2 groups, collecting 2000 samples using a No-U-Turn sampler. The population level parameters are shown in Fig. C.

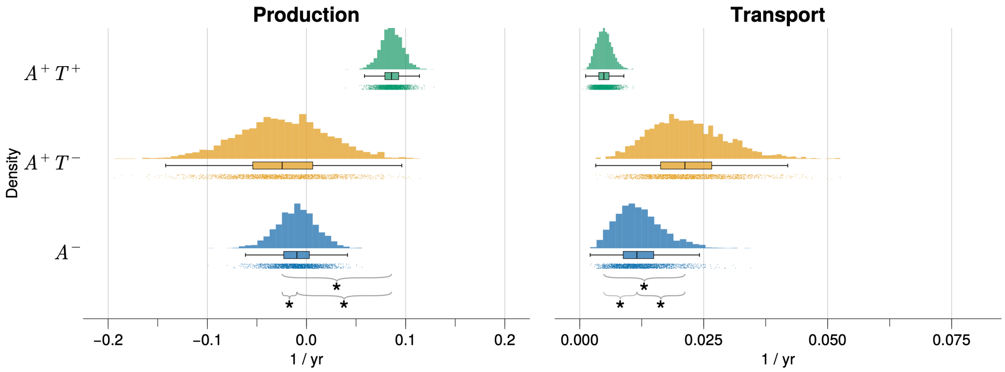

Fig C. Posterior distributions for BF2 and Schaefer atlas. Population-level distributions for A^+T^+, A^+T^- and A^-T^- BF-2 cohorts, using the local FKPP model and the Schaefer-200 atlas. Differences between distributions are tested using a two sample Mann-Whitney U test and significance $p < 0.01$ is denoted by $*$. Data underlying this figure can be found at https://doi.org/10.5281/zenodo.15389493

References

1. Larivière S, Paquola C, yong Park B, Royer J, Wang Y, Benkarim OM, et al. The ENIGMA Toolbox: multiscale neural contextualization of multisite neuroimaging datasets. Nature Methods. 2021;18:698 – 700.
